# Dendritic defect-rich palladium–copper–cobalt nanoalloys as robust multifunctional non-platinum electrocatalysts for fuel cells

**DOI:** 10.1038/s41467-018-06043-1

**Published:** 2018-09-12

**Authors:** Chaozhong Li, Qiang Yuan, Bing Ni, Ting He, Siming Zhang, Yong Long, Lin Gu, Xun Wang

**Affiliations:** 1 College of Chemistry and Chemical Engineering, Guizhou University, Guiyang, Guizhou Province 550025 China; 2 Key Lab of Organic Optoelectronics and Molecular Engineering, Department of Chemistry, Tsinghua University, Beijing, 100084 China; 3 Chinese Academy of Sciences and Beijing National Laboratory for Condensed Matter Physics, Beijing, 100190 China

## Abstract

Recently, the development of high-performance non-platinum electrocatalysts for fuel cell applications has been gaining attention. Palladium-based nanoalloys are considered as promising candidates to substitute platinum catalysts for cathodic and anodic reactions in fuel cells. Here, we develop a facile route to synthesize dendritic palladium–copper–cobalt trimetallic nanoalloys as robust multifunctional electrocatalysts for oxygen reduction and formic acid oxidation. To the best of our knowledge, the mass activities of the dendritic Pd_59_Cu_30_Co_11_ nanoalloy toward oxygen reduction and formic acid oxidation are higher than those previously reported for non-platinum metal nanocatalysts. The Pd_59_Cu_30_Co_11_ nanoalloys also exhibit superior durability for oxygen reduction and formic acid oxidation as well as good antimethanol/ethanol interference ability compared to a commercial platinum/carbon catalyst. The high performance of the dendritic Pd_59_Cu_30_Co_11_ nanoalloys is attributed to a combination of effects, including defects, a synergistic effect, change of *d*-band center of palladium, and surface strain.

## Abstract (web summary)

Fuel cells are promising for sustainable energy generation, but are limited by the performance of electrocatalysts. Here the authors synthesize dendritic palladium–copper–cobalt nanoalloys with electrocatalytic activity for oxygen reduction and formic acid oxidation as well as alcohol tolerance.

## Introduction

Fuel cells (FCs) are efficient, clean, and sustainable energy generation units that produce electricity from fuels such as hydrogen, formic acid, methanol, and ethanol^1–3^. Platinum (Pt) is widely applied as a catalyst for anode and cathode reactions in FCs^4–8^, but many factors, including high cost that accounts for over 55% of the total cost^9^, scarcity, poor durability, and sluggish reaction kinetics of the oxygen reduction reaction (ORR), tremendously impede the commercial application of FCs. Therefore, developing highly effective non-Pt alloy catalysts for FCs has aroused great interest and concern of researchers from all over the world since such alloys can enhance the catalytic performance through optimizing the binding energy between reactants, intermediates and products with the alloy surface at the nanoscale^10–13^. For instance, Linic’s group, Yoo’s group, and Yang’s group, respectively reported Ag–Co alloy, Rh–Sn alloy, Pd–Rh alloy, and Au–Rh alloy catalysts for electrochemical ORR in basic media^14–18^. However, most of the mass activities reported on non-Pt metal catalysts are below 0.20 A mg^−^^1^ for ORR, which is still far from the target (0.44 A mg^−1^_Pt_ at 0.9 V reversible hydrogen electrode (RHE)) of the U.S. Department of Energy^19^. Besides activities, the alcohol (methanol or ethanol) tolerance of metal catalysts is an important concern for direct alcohol (methanol or ethanol) FCs since these fuel molecules generally penetrate the polymer electrolyte membrane and diffuse from the anode to the cathode, which will greatly lower the performance and efficiency of metal catalysts. However, no alcohol tolerance experiments were mentioned on aforementioned non-Pt metal catalysts.

Among non-Pt multimetallic electrocatalysts, Pd-based electrocataysts have received ample attention for organic molecular oxidation and ORR^20–39^. Previous reports have shown that Pd-based nanoalloys can exhibit superior catalytic performance relative to Pt catalysts for FC applications^40–43^. As we know, the size, composition and complex shape (e.g., a dendritic shape) can be used to tune the electrocatalytic performance of Pd-based catalysts. Furthermore, many parameters, such as Pd–Pd interatomic distance, the number of Pd nearest neighbors, the *d*-band center of Pd and the Pd content on the nanoalloy surface, will change when Pd alloys with 3*d* transition metals^10,12,13^. Therefore, rational regulation of these parameters can boost the catalytic activity and/or durability of Pd-based nanocatalysts for ORR and/or small organic molecular oxidation. For instance, Han’s group^40^ and Huang’s group^43^ have, respectively, displayed PdCuCo anisotropic structure and ordered spherical PdCuCo with mass activities at 0.9 V versus RHE of 0.18 and 0.13 A mg^−1^_Pd_ for ORR, but no alcohol tolerance experiments were mentioned. Xing’s group has reported that the mass activity (MA) of palladium–cobalt phosphorus/carbon (Pd–CoP/C) is 2.757 A mg^−1^_Pd_ toward formic acid oxidation (FAO)^21^. Zhang’s group has reported that 4H/face-centered cubic (fcc) Au@Pd core–shell nanorods exhibit high activity toward ethanol oxidation (EO)^24^. However, to the best of our knowledge, there is no report on the synthesis of trimetallic PdCuCo dendritic nanoalloys as robust multifunctional electrocatalysts for both ORR and FAO.

Herein, we report a facile synthesis of trimetallic PdCuCo dendritic nanoalloys with abundant defects. The electrocatalytic performance of these as-synthesized dendritic PdCuCo nanoalloys toward ORR and FAO has been investigated. Compared with commercial Pt/C or Pd black, the dendritic Pd_59_Cu_30_Co_11_ nanoalloys demonstrate much higher MA and durability. Besides, the dendritic Pd_59_Cu_30_Co_11_ nanoalloys also exhibit high alcohol (methanol or ethanol) tolerance compared with commercial Pt/C. Thus, this approach provides an effective route for fabricating dentritic PdCuCo nanoalloys as robust multifunctional electrocatalysts for ORR and FAO by creating abundant defects.

## Results

### Structural characterization

**Fig. 1:**
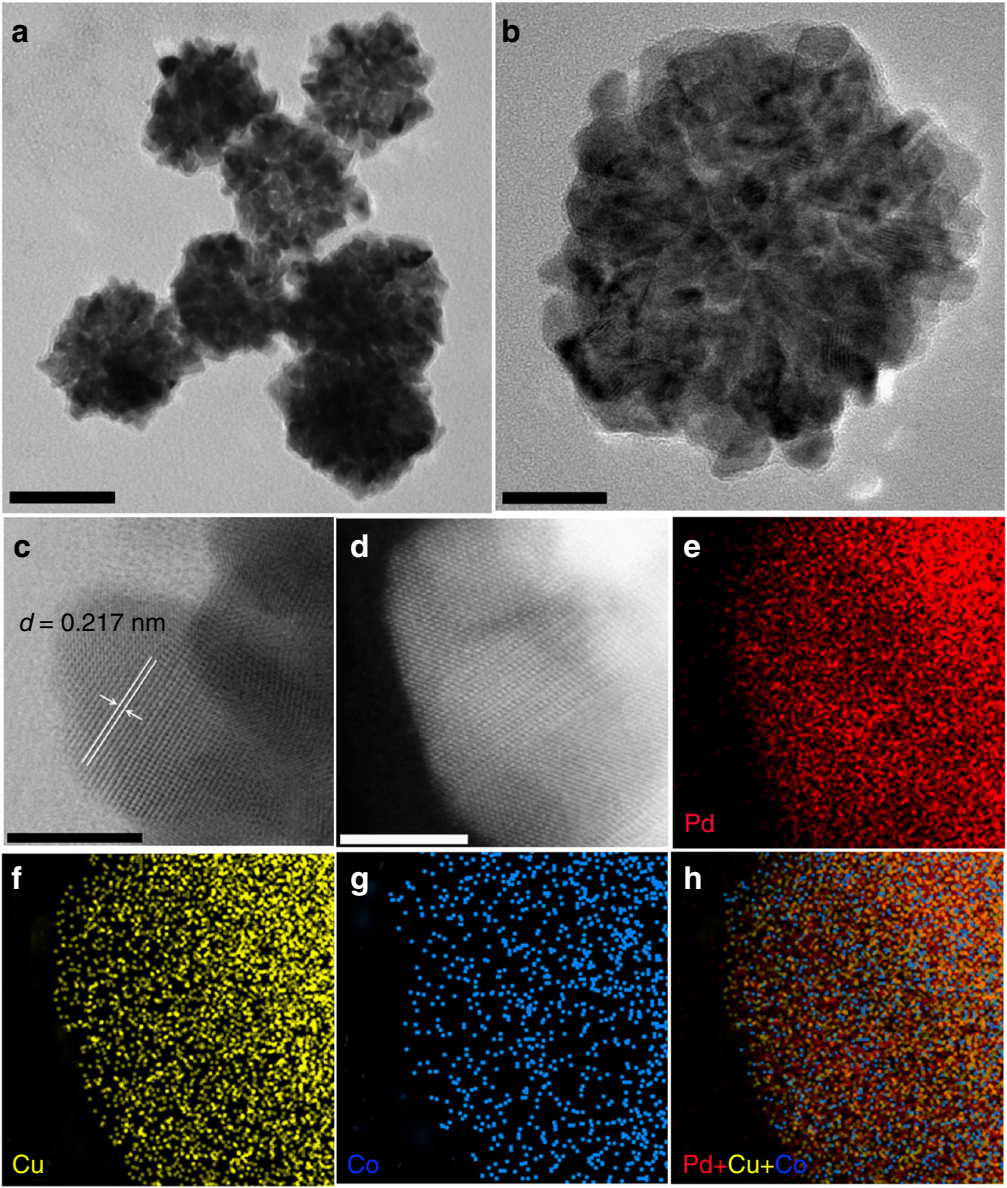
Characterization of the as-synthesized Pd_59_Cu_30_Co_11_ nanoalloys. The typical transmission electron microscopy (TEM) (**a**, **b**), the high-resolution TEM (HRTEM) (**c**) images, the aberration-corrected high-resolution high-angle annular-dark field scanning transmission electron microscopy (HAADF-STEM) (**d**) images and the corresponding energy dispersive X-ray spectroscopy (EDS) elemental mapping (**e**–**h**) images of the as-synthesized Pd_59_Cu_30_Co_11_ nanoalloys. (Scale bar: **a** is 50 nm, **b** is 20 nm, **c,** **d** are 5 nm.)

**Fig. 2:**
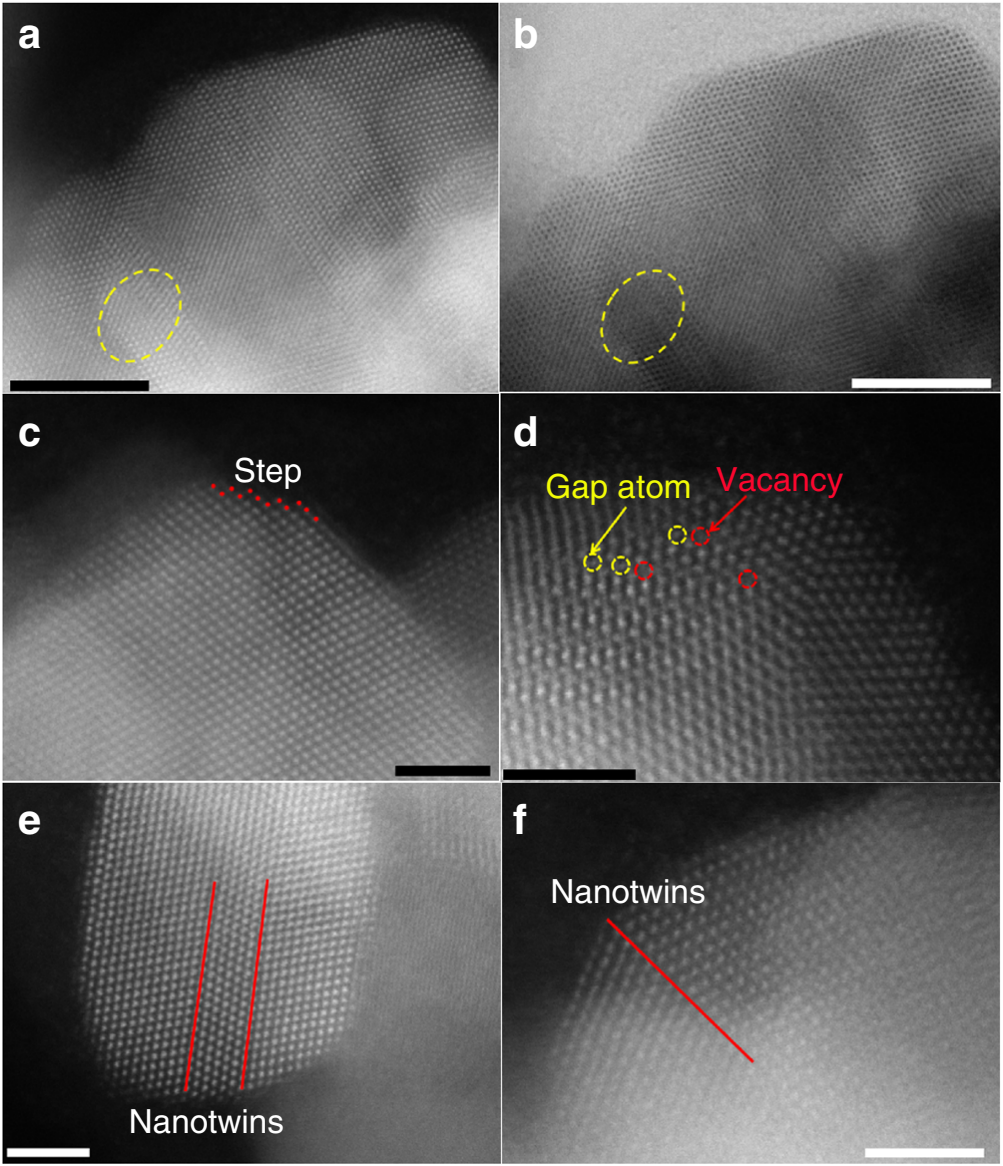
Surface defect characterization of the as-synthesized Pd_59_Cu_30_Co_11_ nanoalloys. Defect analysis images of the as-synthesized dendritic Pd_59_Cu_30_Co_11_ nanoalloys through aberration-corrected transmission electron microscopy (TEM). **a**, **b** Edge, corner and lattice disorder (in yellow dashed circle); **c** step; **d** gap atom in yellow dashed circle and vacancy in red dashed circle; **e**, **f** Nanotwins. (Scale bar: **a**, **b** are 5 nm, **c**–**f** are 2 nm.)

Figure 1a, b and Supplementary Fig. 1a showed the representative transmission electron microscopy (TEM) images of the as-synthesized Pd_59_Cu_30_Co_11_ nanocrystals (the composition was determined by the inductively coupled plasma optical emission spectrometry (ICP–OES), Supplementary Table 1). As can be seen, the obtained products were of uniform size of 66.25 ± 4.5 nm (Supplementary Fig. 1b) and displayed dendritic shapes. These dendritic particles were assembled with dozens of small grains, and the size of most small grains was sub-5.0 nm, around 4.28 nm (Supplementary Fig. 1c). Well-resolved lattice fringes (Fig. 1c) are observed in the sub-5.0 nm grains and the lattice distance of 0.217 nm is very closed to the (111) interplanar distance of face-centered cubic (*fcc*) Pd. The energy dispersive X-ray spectroscopy data of one single dendritic particle showed that the as-synthesized dendritic particle consists of Pd, Cu, and Co (Supplementary Fig. 1d). The X-ray diffraction (XRD) patterns (Supplementary Fig. 1e) of the as-synthesized Pd_59_Cu_30_Co_11_ nanocrystals showed four peaks located at 40.45, 47.05, 68.70, and 81.85° 2*θ* that can be indexed to (111), (200), (220), and (311) planes of the *fcc* Pd (JCPDS-46-1403)^44^, respectively. The diffraction pattern is not characteristic of *fcc* Cu (JCPDS-04-0836)^23^ and *fcc* Co (JCPDS-15-0806)^45^ phases. The peak positions of Pd_50_Cu_50_, Pd_88_Co_12_, and Pd_59_Cu_30_Co_11_ are shifted to higher angles relative to those of the pure Pd crystal (JCPDS-46-1403), indicating that the smaller Cu and Co atoms are incorporated into the Pd lattice. According to the XRD patterns and the Debye–Scherrer equation^46^ (Table 1), Cu and/or Co entering the Pd lattice can induce lattice contraction and strain variation, and the strain variations of trimetallic PdCuCo nanoalloys (Pd_59_Cu_30_Co_11_:3.50%; Pd_56_Cu_38_Co_6_:2.56%; Pd_62_Cu_16_Co_22_:2.11%) are higher than that of bimetallic Pd_50_Cu_50_ (1.62%) and Pd_88_Co_12_ (0.72%), which indicates that simultaneously introducing Cu and Co atoms should result in greater strain variation than introducing only Cu or Co in current system and would enhance catalytic performance of nanocrystals^12,13,39,40,43^. The results of XRD indicated the formation of Pd, Cu, and Co nanoalloys. Moreover, the nanoalloy structure of the as-synthesized Pd_59_Cu_30_Co_11_ nanodendrites was further confirmed by aberration-corrected high-resolution elemental mapping analysis (Fig. 1d–h). The elemental mapping of Pd_59_Cu_30_Co_11_, showed that the Pd, Cu, and Co distributed throughout the whole particle (Fig. 1e–h). Simultaneously, the Pd, Cu, and Co atoms were verified to coexist in the topmost atomic layer within near-surface of the as-synthesized Pd_59_Cu_30_Co_11_ nanodendrite and Pd atoms neighbored Cu and Co atoms. The aberration-corrected high-resolution TEM was further used to analyze the surface structure of the as-synthesized Pd_59_Cu_30_Co_11_ nanodendrite. As shown in Fig. 2, abundant defects including low-coordination number (edges, corners, and steps) atoms, grain boundaries, lattice disorder, gap atoms, vacancies, and nanotwins were clearly observed in the surface. These defects have been confirmed to act as highly active sites and can boost the catalytic performance of catalysts in catalytic reaction^4,19,47–51^.

**Table 1:** The X-ray diffraction (XRD) results of different samples

Samples	2*θ*/degree (111)	Lattice parameter (1/Å)	Strain (%)
Pd crystal (JCPDS-46-1403)^23^	40.11	0.2226	–
Pd_50_Cu_50_	41.68	0.2262	1.62
Pd_88_Co_12_	40.15	0.2242	0.72
Pd_56_Cu_38_Co_6_	40.49	0.2283	2.56
Pd_59_Cu_30_Co_11_	40.21	0.2304	3.50
Pd_62_Cu_16_Co_22_	40.19	0.2273	2.11

**Fig. 3:**
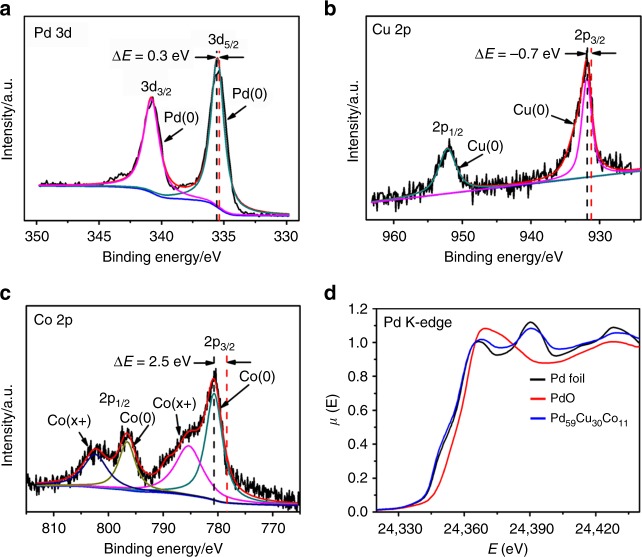
In-depth composition and structural analyses of the Pd_59_Cu_30_Co_11_ nanoalloys. The X-ray photoelectron spectroscopy (XPS) spectra of the as-synthesized dendritic Pd_59_Cu_30_Co_11_ nanoalloys **a** Pd 3*d* region, **b** Cu 2*p* region, **c** Co 2*p* region (the red dash lines in pictures indicate the standard positions of Pd 3*d*, Cu 2*p* and Co 2*p*), **d** the X-ray absorption near edge structure (XANES) spectra for the Pd K-edge

X-ray photoelectron spectroscopy (XPS) is generally accepted to analyze the surface composition and state of nanocrystal surface within 2.0 nm. The XPS results (Fig. 3) of the as-synthesized Pd_59_Cu_30_Co_11_ showed that the Pd, Cu, and Co atoms coexisted in the near-surface of Pd_59_Cu_30_Co_11_nanoalloys. The binding energy of the Pd 3*d* peaks (Fig. 3a) shifted to 335.5 and 340.7 eV compared to the standard Pd 3*d* peaks (335.2 and 340.4 eV), and the binding energy of metallic Pd in the nanosurface of Pd_59_Cu_30_Co_11_ nanoalloys positively shifted about 0.3 eV, which meant the downshift of the *d*-band center of Pd and this result was in accordance with aforementioned reports^13,46,52,53^. To further probe the local structure of the Pd_59_Cu_30_Co_11_ nanoalloys at atomic scale, the X-ray absorption fine structure measurements were performed at the Pd K-edge. From the X-ray absorption near edge structure of the Pd K-edge (Fig. 3d), we can clearly see that the Pd K-edge curve of the Pd_59_Cu_30_Co_11_ nanoalloys was almost the same as that of metal state of Pd-foil and was quite different from that of the PdO, indicating that the as-synthesized Pd_59_Cu_30_Co_11_ did not contain the PdO. The binding energy of Cu 2*p* peaks (Fig. 3b) negatively shifted to 931.9 and 951.9 eV compared to the standard Cu 2*p* peaks (932.6 and 952.56 eV). The binding energy of Co 2*p* peaks (Fig. 3c) showed a positive shift compared with the standard metallic Co peaks (778.3 and 793.2 eV), and the oxidized state Co also existed in the nanosurface of Pd_59_Cu_30_Co_11_ nanoalloys. The shifts of binding energy could be ascribed to the strong electronic effects between Pd, Cu, and Co elements through *d*-band hybridization upon alloying within the atomic range^12,13,40,41^.

### ORR performance

**Fig. 4:**
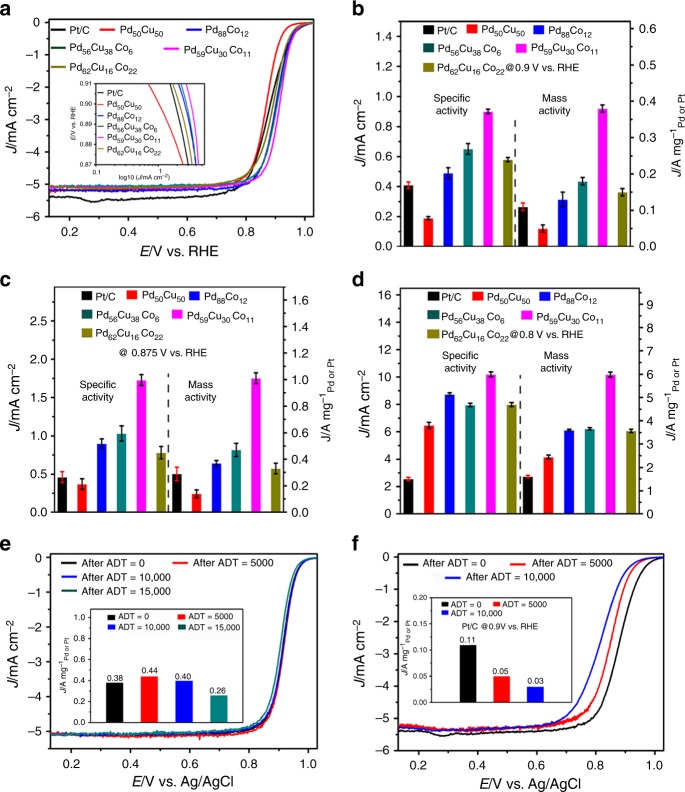
Oxygen reduction reaction performance characterization. The oxygen reduction reaction (ORR) performance of Pt/C, Pd_88_Co_12_, Pd_50_Cu_50_, Pd_56_Cu_38_Co_6_, Pd_59_Cu_30_Co_11_, and Pd_62_Cu_16_Co_22_ catalysts. **a** The ORR polarization curves were recorded in O_2_-saturated 0.1 mol L^−1^ (M) potassium hydroxide (KOH) and the inset was the corresponding specific activity Tafel plots. Histograms of ORR specific activities and mass activities of the Pt/C, Pd_88_Co_12_, Pd_50_Cu_50_, Pd_56_Cu_38_Co_6_, Pd_59_Cu_30_Co_11_, and Pd_62_Cu_16_Co_22_ catalysts at 0.9 V (**b**), 0.875 V (**c**), and 0.8 V (**d**) versus reversible hydrogen electrode (RHE). **e**, **f** The ORR polarization curves for the Pd_59_Cu_30_Co_11_ and commercial Pt/C catalysts before and after cycles of accelerated durability tests (ADTs) (the inset was the corresponding mass activity at 0.90 V versus RHE). Error bars are ±s.d.

**Fig. 5:**
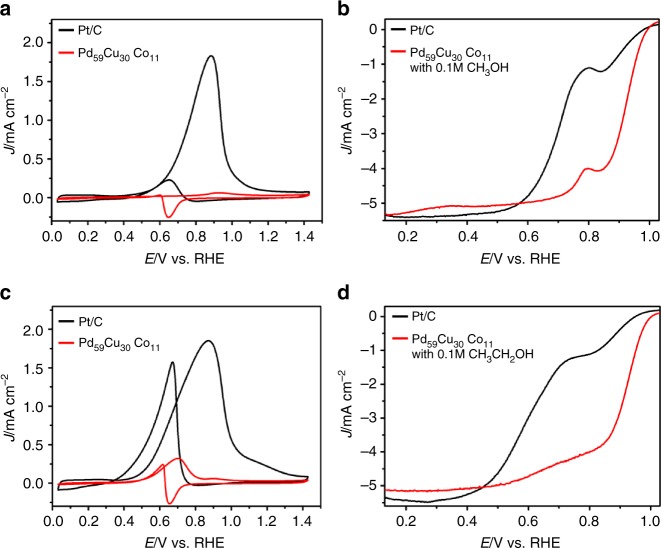
Methanol and ethanol tolerance tests. Cyclic voltammetry (CV) curves of the dendritic Pd_59_Cu_30_Co_11_ nanoalloys and the commercial Pt/C in N_2_-saturated 0.1 mol L^−1^ (M) potassium hydroxide (KOH) + 0.1 M methanol (CH_3_OH) (**a**) or ethanol (CH_3_CH_2_OH) (**c**) solution at 50 mV s^−1^. Oxygen reduction reaction (ORR) polarization curves of the dendritic Pd_59_Cu_30_Co_11_ nanoalloys and the commercial Pt/C in O_2_-saturated 0.1 M KOH + 0.1 M methanol (**b**) or ethanol (**d**) solution at 10 mV s^−1^ and rotation rate of 1600 rpm

The ORR performance of the dendritic Pd_59_Cu_30_Co_11_ nanoalloys have been investigated compared with commercial Pt/C, bimetallic Pd_50_Cu_50_ and Pd_88_Co_12_ and trimetallic Pd_56_Cu_38_Co_6_ and Pd_62_Cu_16_Co_22_ alloy nanodendrites (Supplementary Fig. 2, Supplementary Fig. 3, Supplementary Fig. 4, Supplementary Fig. 5, and Supplementary Table 1) obtained at the same synthetic method as the dendritic Pd_59_Cu_30_Co_11_ nanoalloys in an O_2_-saturated 0.1 mol L^−1^ (M) KOH solution with a scan rate of 10 mV s^−1^ and a rotation rate of 1600 pm at room temperature. The ORR polarization curves of these samples are shown in Fig. 4a. The half-wave potentials of the Pd_50_Cu_50_, Pd_88_Co_12_, Pd_59_Cu_30_Co_11_, Pd_56_Cu_38_Co_6_, Pd_62_Cu_16_Co_22_, and commercial Pt/C catalysts are 0.86, 0.90, 0.91, 0.90, 0.89, and 0.87 V, respectively, which exhibited that the Pd_59_Cu_30_Co_11_ nanoalloys had a greatly reduced ORR overpotential and indicated the superior activity. This was also confirmed by Tafel plots of these samples (Fig. 4a, inset). The positions of hydrogen adsorption/desorption peaks were often used to analyze the surface structure of electrocatalyst (Supplementary Fig. 6a). As can been seen, the hydrogen adsorption/desorption peaks on these samples located at different positon, which meant the surface structure and active site of these electrocatalysts were quite different and would display different ORR performance. The electrochemically active surface area (ECSA), calculated from the results of CO-stripping (Supplementary Fig. 7) of Pd_50_Cu_50_, Pd_88_Co_12_, Pd_59_Cu_30_Co_11_, Pd_56_Cu_38_Co_6_, and Pd_62_Cu_16_Co_22_ dendritic nanoalloys was around 37.9, 41.2, 58.7, 46.3, and 44.7 m^2^ g^−1^_Pd_, respectively. The ECSA of commercial Pt/C was 67.8 m^2^ g^−1^_Pt_. Figure 4b showed the specific activity (SA) and MA of ORR normalized by ECSA and Pd or Pt loading at 0.9 V versus a RHE using Koutecky–Levich equation, respectively. Among them, the Pd_59_Cu_30_Co_11_ nanoalloys showed the best SA and MA. The SA and MA on the Pd_59_Cu_30_Co_11_ nanoalloys were 0.90 mA cm^−2^ and 0.38 A mg^−1^_Pd_, respectively. The MA on Pd_59_Cu_30_Co_11_ nanoalloys was 3.45 times higher than commercial Pt/C catalysts (0.11 A mg^−1^_Pt_). Impressively, our Pd_59_Cu_30_Co_11_ nanoalloys had a competitively high MA in comparison with previously reported non-Pt metal nanocatalysts (Supplementary Table 2) that is also superior to the recently reported PdCuCo anisotropic structure (0.18 A mg^−1^_Pd_)^40^ and spherical PdCuCo (0.13 A mg^−1^_Pd_)^43^. Furthermore, at 0.875 and 0.8 V versus RHE (Fig. 4c, d), the Pd_59_Cu_30_Co_11_ nanoalloys exhibited the largest ORR SA (1.73 mA cm^−2^ at 0.875 V, 3.76 times that of commercial Pt/C; 10.20 mA cm^−2^ at 0.8 V, 3.98 times that of commercial Pt/C) and MA (1.01 A mg^−1^_Pd_ at 0.875 V, 3.48 times that of commercial Pt/C; 5.98 A mg^−1^_Pd_ at 0.8 V, 3.71 times that of commercial Pt/C). Furthermore, in order to determine the electron transfer number on Pd_59_Cu_30_Co_11_ nanoalloys, the ORR polarization curves of Pd_59_Cu_30_Co_11_ nanoalloys were investigated by rotating disk electrode (RDE) measurements at different rotation rates from 400 to 2500 rpm in 0.1 M KOH solution (Supplementary Fig. 8a). The Koutecky–Levich (K–L) plots for Pd_59_Cu_30_Co_11_ nanoalloys calculated at different potentials from 0.4 to 0.8 V were shown in Supplementary Fig. 8b. The slopes were parallel at different potentials, showing the first-order reaction kinetics and the electron transfer number was around four that indicated fully electrocatalytic reduction of oxygen into water on the Pd_59_Cu_30_Co_11_ nanoalloys. The alcohol (methanol or ethanol) tolerance of the dendritic Pd_59_Cu_30_Co_11_ nanoalloys was also investigated for ORR. Before testing ORR, the electrocatalytic activity toward methanol or ethanol of the dendritic Pd_59_Cu_30_Co_11_ nanoalloys and commercial Pt/C were performed using cyclic voltammetry in 0.1 M KOH + 0.1 M methanol/ethanol solution. As shown in Fig. 5a–c, typical methanol and EO peaks were observed at 0.88 and 0.87 V, respectively, on commercial Pt/C, while methanol oxidation peak was hardly generated on the dendritic Pd_59_Cu_30_Co_11_ nanoalloys and EO peak was only one-sixth of that on commercial Pt/C, which meant much lower electrocatalytic activity toward methanol and EO on the dendritic Pd_59_Cu_30_Co_11_ nanoalloys compared with commercial Pt/C. The ORR polarization curves (Fig. 5b–d) showed the commercial Pt/C had a weak methanol and ethanol tolerance capacity. The half-wave potentials of the commercial Pt/C are 0.707 and 0.619 V versus RHE in the presence of methanol and ethanol, respectively, and the half-wave potentials negatively shifted 0.163 and 0.251 V, respectively, compared with that (0.87 V versus RHE; Fig. 4a) without methanol and ethanol. However, the dendritic Pd_59_Cu_30_Co_11_ nanoalloys exhibited excellent methanol and ethanol tolerance capacity. The half-wave potential (0.91 V without methanol and ethanol; Fig. 4a) were barely affected by methanol (0.909 V versus RHE) and ethanol (0.907 V versus RHE). These results showed that the dendritic Pd_59_Cu_30_Co_11_ nanoalloys had much better antimethanol and ethanol interference ability than the commercial Pt/C.

The dendritic Pd_59_Cu_30_Co_11_ nanoalloys also presented long-term durability under the ORR reaction condition. The accelerated durability tests (ADTs) were used to evaluate ORR durability under a sweep rate of 100 mV s^−1^ between 0.53 and 1.03 V in O_2_-saturated 0.1 M KOH solution. After 5000, 10,000, and 15,000 sweeping cycles, the MA of the Pd_59_Cu_30_Co_11_ nanoalloy was 0.44, 0.40, and 0.26 A mg^−1^_Pd_, respectively, at 0.9 V versus RHE (Fig. 4e–f), which first experienced a rise and then a drop. This could be ascribed to the surface structure change under the ADTs and the same phenomenon was also observed about trimetallic PtPdAu alloys reported by Ding’s group^54^. However, the MA of Pt/C after 5000 and 10,000 sweeping cycles was 0.05 and 0.03 A mg^−1^_Pt_, respectively, at 0.9 V versus RHE. After 10,000 sweeping cycles, the Pt/C catalyst shows the 72.7% loss of MA, while the Pd_59_Cu_30_Co_11_ nanoalloys showed no loss of MA (0.40 A mg^−1^_Pd_) that was 13.3 times higher than Pt/C. And after 15,000 sweeping cycles, the MA (0.26 A mg^−1^_Pd_) of the Pd_59_Cu_30_Co_11_ nanoalloys is 2.36 times higher than initial MA (0.11 A mg^−1^_Pt_) of Pt/C and had a loss of 31.6% compared with the initial MA (0.38 A mg^−1^_Pd_). The above results exhibited that the Pd_59_Cu_30_Co_11_ nanoalloys had considerable enhancement with respect to durability compared with commercial Pt/C. After ADTs, we checked the morphologies of the Pd_59_Cu_30_Co_11_ nanoalloys using TEM (Supplementary Fig. 9). The TEM images showed the Pd_59_Cu_30_Co_11_ nanoalloys also maintained dendritic structure, however, after 15,000 sweeping cycles, the size of the small grains obviously increased due to the movement, aggregation and Ostwald ripening processes under the ADTs^19^, and we can clearly observe that some dendritic nanocrystals are hollow-like shapes, which caused by the electrochemical dealloying of Cu or Co (Supplementary Fig. 9), these were the reason why the catalytic activity decreased. After ADT cycles, the composition of Pd_59_Cu_30_Co_11_ nanoalloys changed (Supplementary Fig. 10). After 5000 cycles, the composition was 69.41 at% Pd, 29.29 at% Cu, and 1.30 at% Co. After 10,000 cycles, the composition was the 77.30 at% Pd, 21.78 at% Cu, and 0.92 at% Co, and after 15,000 cycles it was 83.68 at% Pd, 15.68 at% Cu and 0.64 at% Co. Moreover, the dendritic structure had stronger antiaggregation capacity due to their free-standing architectural feature than commercial Pt/C. For commercial Pt/C, after 10,000 cycles, the size of Pt particles obviously increased relative to initial size, namely, the size increased from sub-3.0 nm to around 10 nm (Supplementary Fig. 11), which led to the sharp fall of activity of commercial Pt/C.

### Formic acid oxidation performance

**Fig. 6:**
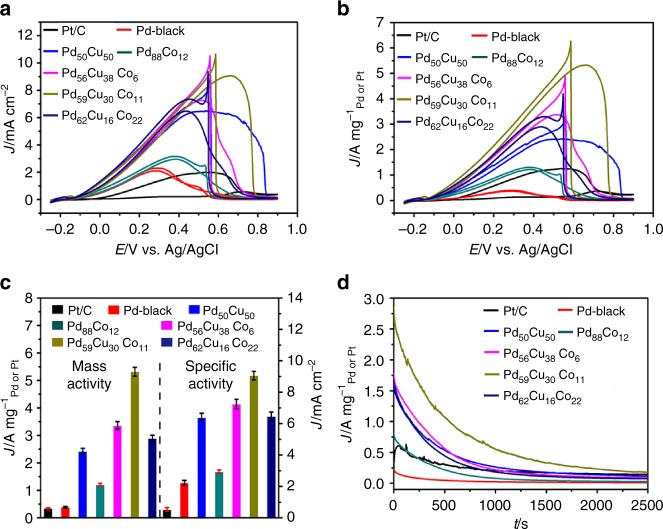
Formic acid oxidation (FAO) characterization. Cyclic voltammetry (CV) curves (specific activity (**a**) and mass activity (**b**)) of the as-synthesized Pd_50_Cu_50_, Pd_88_Co_12_, Pd_56_Cu_38_Co_6_, Pd_59_Cu_30_Co_11_, Pd_62_Cu_16_Co_22_, commercial Pt/C, and Pd black with a scan rate of 50 mV s^−1^ in 0.1 mol L^−1^ (M) perchloric acid (HClO_4_) + 0.5 M formic acid (HCOOH) solution at room temperature. **c** Histograms of formic acid oxidation (FAO) specific activity and mass activity. Error bars are ±s.d. **d** Current–time curves of the as-synthesized Pd_50_Cu_50_, Pd_88_Co_12_, Pd_56_Cu_38_Co_6_, Pd_59_Cu_30_Co_11_, Pd_62_Cu_16_Co_22_, commercial Pt/C, and Pd black recorded at 0.25 V for 2500 s in 0.1 M HClO_4_ + 0.5 M HCOOH solution at room temperature

The dendritic Pd_59_Cu_30_Co_11_ nanoalloys also exhibited excellent FAO activity. Figure 6a showed that the SA of the Pd_50_Cu_50_, Pd_88_Co_12_, Pd_56_Cu_38_Co_6_, Pd_59_Cu_30_Co_11_, Pd_62_Cu_16_Co_22_, commercial Pd black, and Pt/C were around 6.38, 2.94, 7.25, 9.06, 6.46, 2.24, and 0.56 mA cm^−2^, respectively. The MA (Fig. 6b) of the Pd_50_Cu_50_, Pd_88_Co_12_, Pd_56_Cu_38_Co_6_, Pd_59_Cu_30_Co_11_, Pd_62_Cu_16_Co_22,_ commercial Pd black, and Pt/C was 2.42, 1.21, 3.36, 5.32, 2.89, 0.39 A mg^−1^_Pd_ and 0.35 A mg^−1^_pt_, respectively. These values (Fig. 6c) showed that the as-synthesized Pd-based alloys displayed greatly enhanced activity compared with commercial Pd black and Pt/C. And the values of Pd_59_Cu_30_Co_11_ nanoalloys were the largest. The FAO SA and MA of the Pd_59_Cu_30_Co_11_ nanoalloys were 4.04 and 13.6 times higher than that of commercial Pd black, respectively, and 16.1 and 15.2 times higher than commercial Pt/C, respectively. Similar to the case of ORR, the Pd_59_Cu_30_Co_11_ nanoalloys display high MA relative to previously reported Pd-based electrocatalysts (Supplementary Table 3). Moreover, the stability of the as-synthesized PdCuCo nanoalloys was evaluated by current–time curves and showed better stability than Pd_50_Cu_50_, Pd_88_Co_12_, commercial Pd black and Pt/C (Fig. 6d).

## Discussion

The excellent electrocatalytic performance of the as-synthesized dendritic Pd_59_Cu_30_Co_11_ nanoalloys is mainly ascribed to the following reasons: (1) The special dendritic nanostructure of the Pd_59_Cu_30_Co_11_ nanoalloy can provide abundant defects, such as low-coordination number atoms (edges, corners, and steps), grain boundaries, lattice disorder, nanotwins, gap atoms, and vacancies, which have been confirmed to boost the catalytic performance in catalytic reactions^4,19,43,47–51^. (2) Synergistic surface effects between Pd, Cu, and Co elements are present in the topmost atomic layer and favor the removal of poisoning intermediates (such as CO adsorbed (CO_ads_) and OH adsorbed (OH_ads_)) that are produced in the electrocatalytic reaction^6,23,37,42^. Moreover, metallic Cu and Co have been found to constitute sites for activating oxygen, water or small organic molecules and improve the activity and durability in FCs^13,23,54–62^. (3) Pd alloying with Cu and Co changes the surface electronic structure through ligand effect and downshifts the *d*-band center of Pd, which can tune the adsorption/desorption capacities of activating reactant molecules or intermediates and enhance the catalytic performance^39,40,43^. (4) Strain effects have also been shown to contribute to high catalytic performance^7,10,12,60–62^. The surface strain in dendritic Pd_59_Cu_30_Co_11_ nanoalloys may be attributed to the lattice mismatch triggered by the incorporation of smaller Cu and Co atoms into the Pd *fcc* lattice, small size of sub-5.0 nm grains that construct the dendritic structure and a boundary that originates from the mutual cross-linking of small grains^59,60^.

In conclusion, the defect-rich dendritic PdCuCo nanoalloys have been synthesized by a facile one-pot method to be developed as high-performance non-Pt nanocatalysts toward ORR and FAO. Among them, a Pd_59_Cu_30_Co_11_ nanoalloy shows the best catalytic performances. For ORR, the SA (0.90 mA cm^−2^ at 0.9 V and 1.73 mA cm^−2^ at 0.875 V versus RHE) and MA (0.38 A mg^−1^_Pd_ at 0.9 V versus RHE and 1.01 A mg^−1^_Pd_ at 0.875 V versus RHE) of the dendritic Pd_59_Cu_30_Co_11_ nanoalloy are 2.19 and 3.76 times and 3.45 and 3.48 times those of commercial Pt/C, respectively. The long-term durability has been evaluated through an ADT. After 10,000 cycles, the commercial Pt/C catalyst shows the 72.7% loss of MA, while the Pd_59_Cu_30_Co_11_ nanoalloy showed no loss of MA. After 15,000 cycles, the MA (0.26 A mg^−1^_Pd_) of the Pd_59_Cu_30_Co_11_ nanoalloy is 2.36 times higher than initial MA (0.11 A mg^−1^_Pt_) of Pt/C. For FAO, the SA and MA of the Pd_59_Cu_30_Co_11_ nanoalloy is 4.04 and 13.6 times higher, respectively, than commercial Pd black and 16.1 and 15.2 times higher, respectively, than commercial Pt/C. The current–time curves show that the Pd_59_Cu_30_Co_11_ nanoalloy exhibits better stability than commercial Pd black and Pt/C. And the dendritic Pd_59_Cu_30_Co_11_ nanoalloy has good methanol/ethanol tolerance ability. Thus, this work provides an example of the development of a new type of robust multifunctional, non-Pt catalysts for FCs.

## Methods

### Synthesis of dendritic Pd_59_Cu_30_Co_11_ nanoalloys

The dendritic Pd_59_Cu_30_Co_11_ nanoalloys were prepared by using precursors of Na_2_PdCl_4_, CuCl_2_, and CoCl_2_. Firstly, the 0.25 mL of 0.1 M Na_2_PdCl_4_, 0.125 mL of 0.1 M of 0.1 M of CuCl_2_, and 0.125 mL of 0.1 M of CoCl_2_ were added to 8.8 mL of ethylene glycol containing 0.050 g polyvinyl pyrrolidone (PVP-8000), and stirred for 1–2 min then the yellow clear solution was obtained. Secondly, the 0.2 mL ammonium hydroxide was added to the above solution, and stirred for 30 min. Then, the resulting clear solution was transferred to a 15-mL Teflon-lined stainless-steel autoclave. The oven was then heated at 150 °C for 8 h, then the Teflon-lined stainless-steel autoclave was taken out after it was cooled to room temperature. The products were separated several times by centrifugation and washing cycles at 10,000 rpm for 15 min with ethanol. The Pd_59_Cu_30_Co_11_ was redispersed in ethanol. By adjusting the ratio of Cu or Co precursors, the Pd_50_Cu_50_, Pd_88_Co_12_, Pd_56_Cu_38_Co_6_, and Pd_62_Cu_16_Co_22_ nanoalloys can be obtained under the same procedure. Further details are provided in the Supplementary Methods.

### The ORR electrochemical measurement

For the ORR electrochemical measurement, a glassy carbon RDE (PINE, USA) was used. A Hg/HgO (0.1 M KOH) electrode and Pt black plate were used as reference and counter electrodes, respectively. The ORR was performed in the O_2_-saturated 0.1 M KOH solution at room temperature. Then, using a glassy carbon RDE at a rotation rate of 1600 rpm, the potential was scanned from 0.13 to 1.03 V (versus RHE) at a scan rate of 10 mV s^−1^ and were corrected for capacitive currents in N_2_ saturation. The kinetic current (*i*_k_) can be derived from the experimental data using the well-known Koutecky–Levich equation: (1/*i*_K_ = 1/*i* − 1/*i*_L_) at 0.90 V versus RHE, where *i*_L_ and *i* are the diffusion limiting current and measured current (0.90 V versus RHE) at kinetic-diffusion control region, respectively.

The tolerance test: A Hg/HgO (0.1 M KOH) electrode and Pt black plate were used as reference and counter electrodes, respectively. Then MOR or EOR tested with a scan rate of 50 mV s^−1^ at room temperature in 0.1 M KOH + 0.1 M CH_3_OH or 0.1 M KOH + 0.1 M CH_3_CH_2_OH, and the potential was scanned from 0.03 to 1.43 V (versus RHE), after this, using RDE at a rotation rate of 1600 rpm, and the potential was scanned from 0.13 to 1.03 V (versus RHE) at a scan rate of 10 mV s^−1^.

## Electronic supplementary material

Supplementary Information

## Data Availability

All relevant data are available from the corresponding author upon reasonable request.
