# Supplementary material for: Dendritic defect-rich palladium–copper–cobalt nanoalloys as robust multifunctional non-platinum electrocatalysts for fuel cells
Source: Nat Commun. 2018 Sep 12;9:3702. doi: 10.1038/s41467-018-06043-1 (PMC6135778; doi:10.1038/s41467-018-06043-1)
Supplement: Supplementary file 1 — Supplementary Information [file 41467_2018_6043_MOESM1_ESM.pdf]

Supplementary Information

Dendritic defect-rich palladium-copper-cobalt nanoalloys as robust multifunctional non-platinum electrocatalysts for fuel cells

C. Li et al.

Supplementary Methods

Chemicals. Palladium(II) chloride ($PdCl_2$, 99.9%), Sodium(I) chloride (NaCl, 99.99%), Cobalt(II) chloride hexahydrate ($CoCl_2 \cdot 6H_2O$, AR), Polyvinyl Pyrrolidone (PVP-8000), Copper(II)Chloride Dihydrate ($CuCl_2 \cdot 2H_2O$, 99.0%), were purchased from Sigma-Aldrich. ethylene glycol (EG, 99.0%), ammonium hydroxide ($NH_3 \cdot H_2O$, 25-28%), Perchloric acid (G.R.) and absolute ethanol were purchased from the China Medicine Shanghai Chemical Reagent Corp. Commercial Pt/C (20 wt%) and Pd black catalysts were purchased from Johnson Matthey. Chemicals were used as received without further purification. Super pure water (18.25 MΩ cm) was used as the solvent. 0.1 M of Na_2PdCl_4 aqueous solution was prepared by adding 0.235g (4.0 mmol) of NaCl and 0.354g (2.0 mmol) of $PdCl_2$ into 20 mL of water and stirring for 40 minutes, then, the solution was stored without stirring for one day. 0.1 M of $CuCl_2$ aqueous solution was prepared by adding 0.344g $CuCl_2 \cdot 2H_2O$ into 20 mL of water and stirring for 10 minutes. 0.1 M of $CoCl_2$ aqueous solution was prepared by adding 0.476g into 20 mL of water and stirring for 10 minutes.

Electrochemical experiment. All of the electrochemical measurements were performed in a conventional three-electrode cell using a CHI 760e electrochemical analyzer (CH Instruments, Inc., Shanghai). A Ag/AgCl electrode and platinum plate were used as reference and counter electrodes, respectively. The FAO working electrode is a glassy carbon (GC, d=4 mm) electrode embedded into a Teflon holder. Before the electrochemical test, we use alumina powder of size 1.5, 1.0 and 0.05 μm to polish the GC electrode, and use ultrapure water clean it in an ultrasonic bath. The suspension of catalysts was spread on the GC electrode. As soon as the electrode was dried under infrared lamp, 4 μL Nafion diluents (1 wt % Nafion® solution) was coated onto the electrode surface.

The cyclic voltammograms (CVs) were obtained in N_2-saturated 0.1 M $HClO_4$ solution, and the potential was scanned from – 0.25 to 0.9 V (Ag/AgCl) at a scan rate 50 mV s^{-1}. The scan was repeated several times to ensure that a stable cyclic voltammetry (CV) was obtained.

The electrochemically active surface area （ECSA）tests was estimated by CO stripping test: All working electrodes consisted of catalysts were carried out by firstly in the a CO-saturated 0.1 M $HClO_4$ solution electrolytic cell, and let the catalyst adsorb CO at 0.20 V for 300 seconds. Then transfered the working electrodes into N_2-saturated 0.1 M $HClO_4$ solution electrolytic cell to test from 0.0 to 1.1 V (Ag/AgCl) at a scan rate of 50 mV s^{-1}. The ECSA was calculated from the charge involved in the CO adsorption processes using the following equation:

$$ECSA = Q/(0.42 \times M),$$

where Q (mC) is the charge for the CO adsorption. *0.42* (mC/cm^2) is the electrical charge associated with full monolayer adsorption of CO on Pd. The average values and related errors, for the ECSA, specific activity and mass activity, were obtained from the results based on the measurement of more than 5 electrodes made of each catalyst.

Characterizations. Transmission electron microscope (TEM) was characterized on a Hitachi H-7700 at 100KV. A Tecnai G2 F20 S-Twin high-resolution transmission electron microscope at 200KV (HRTEM) was used to characterize the sample. A high resolution aberration corrected transmission electron microscope JEOL-ARM200F, operated at 200kV, was used to perform high-angle annular-dark-field (HAADF) and annular-bright-field (ABF)-STEM and energy-dispersive X-ray spectroscopy (EDS) mapping. X-ray diffraction (XRD) patterns of the samples were recorded on a Bruker D8 Advance diffractometer with CuK_a radiation (l=1.5418Å) with graphite monochromator (40 KV, 40 mA). X-ray photoelectron spectroscopy (XPS) measurements were performed using a PHI Quantum 2000 Scanning ESCA Microprobe (Physical Electronics, USA), using Al K_α X-ray radiation (1486.6 eV) for excitation. Binding energies were corrected from charge effects by reference to the C1s peak of carbon at 284.8 eV. The inductively coupled plasma optical emission spectrometry (ICP-OES) analysis of samples was performed on IRIS Intrepid II XSP (ThermoFisher). X-ray photoelectron spectroscopy on PHI Quantera SXM.

The X-ray Absorption Fine Structure (XAFS). The X-ray absorption find structure spectra data (Pd K-edge) were obtained at BL14W1 station in Shanghai Synchrotron Radiation Facility (SSRF, operated at 3.5 GeV with a maximum current of 250 mA). The data were obtained at room temperature (Pd K-edge in transmission mode). Graphite powders were used as a binder to pelletize samples into disks with a diameter of 13 mm and a thickness of 1mm.

Supplementary Figures

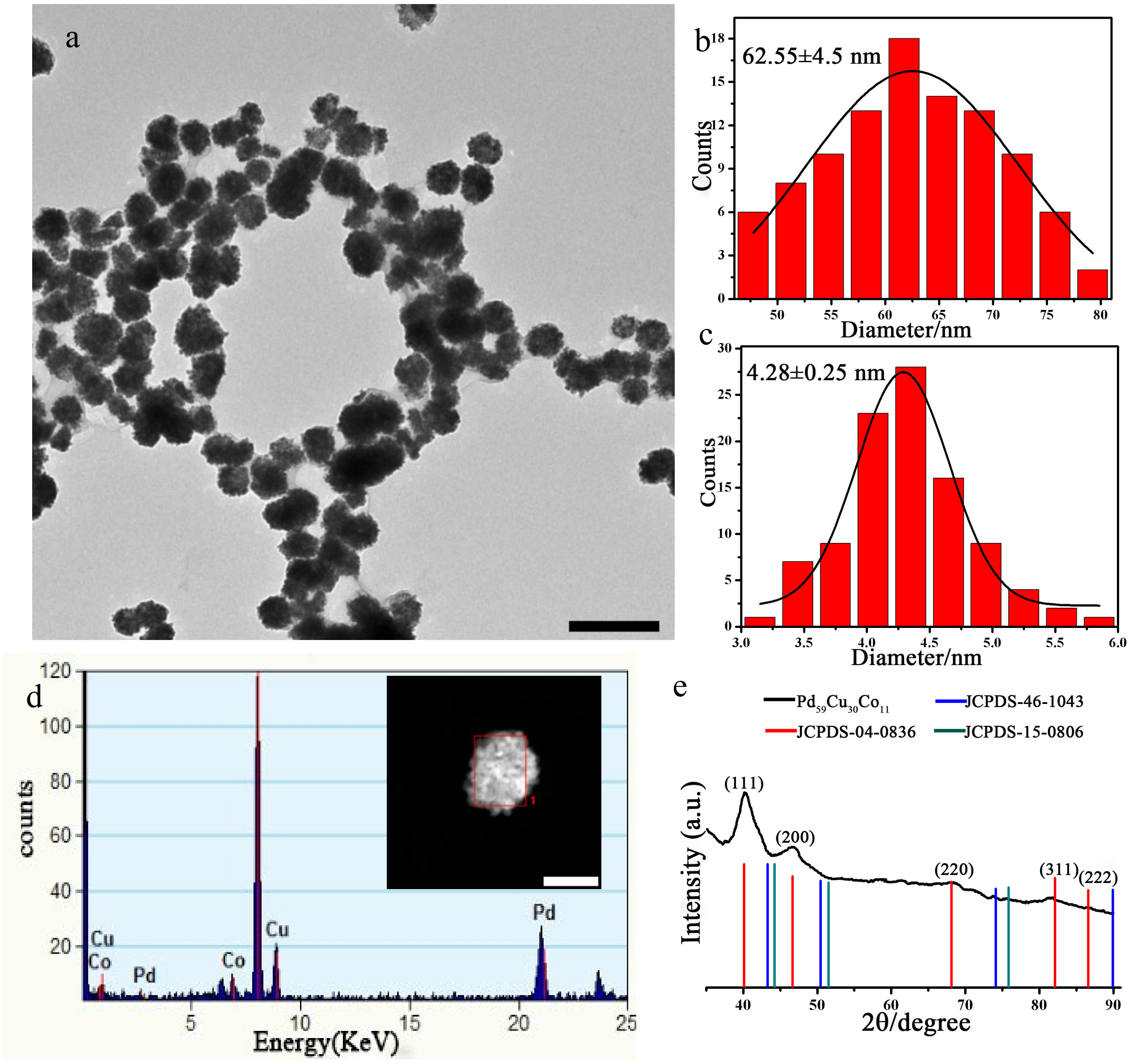

Supplementary Figure 1 | (a) The transmission electron microscopy (TEM) image of the as-synthesized $Pd_{59}Cu_{30}Co_{11}$ nanocrystals. Histogram of size distribution of as-synthesized $Pd_{59}Cu_{30}Co_{11}$ nanodendrites (b) and (c) small particles that assembled the $Pd_{59}Cu_{30}Co_{11}$ nanodendrites. (d) The energy dispersive X-ray spectra (EDS) of one single $Pd_{59}Cu_{30}Co_{11}$ nanodendrite. (e) The X-ray diffraction (XRD) pattern of as-synthesized $Pd_{59}Cu_{30}Co_{11}$ nanodendrites. (Scale bar: a is 200 nm, d inset is 50 nm.)

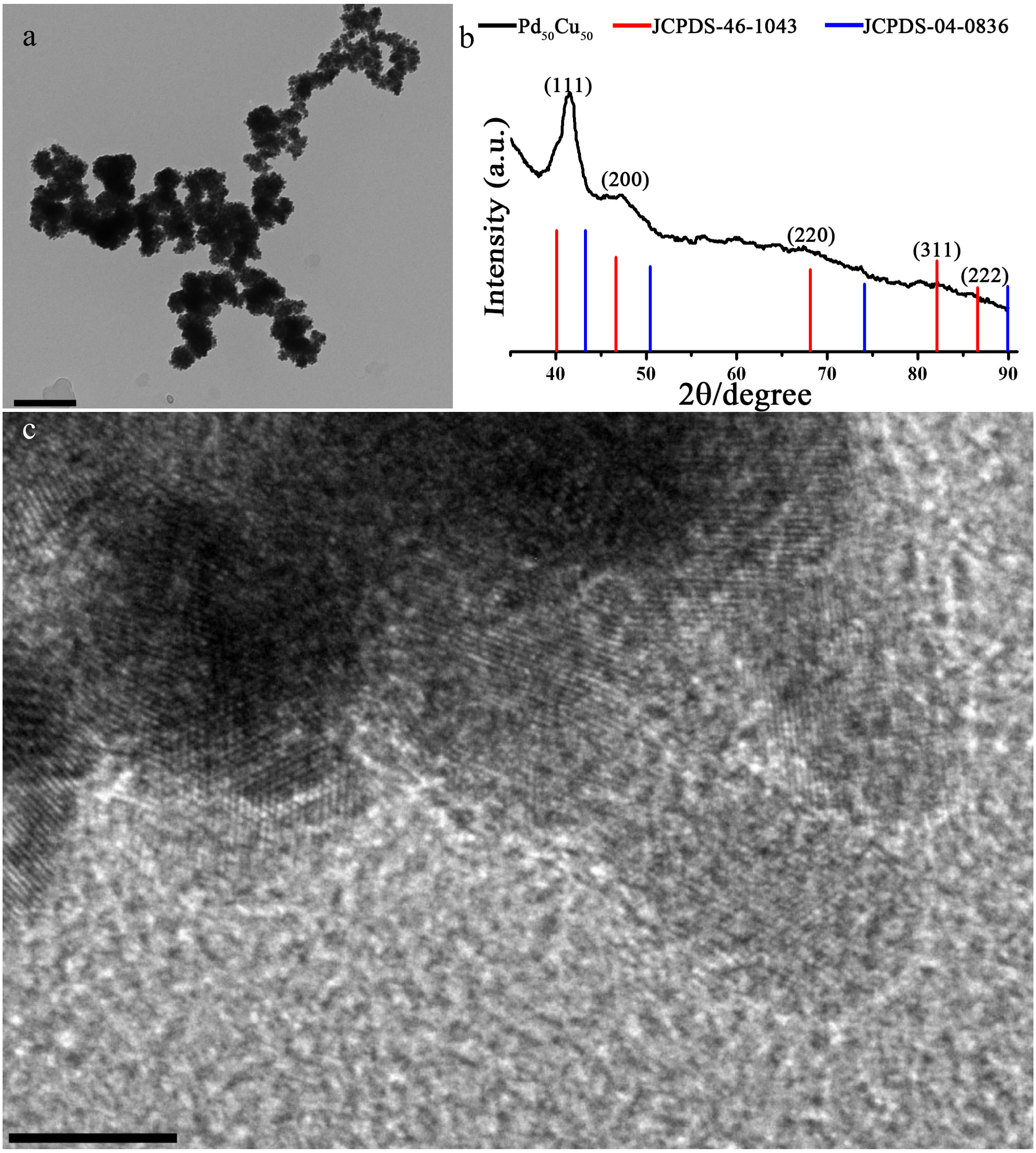

Supplementary Figure 2 | The transmission electron microscopy (TEM) image (a), The X-ray diffraction (XRD) pattern (b) and high-resolution TEM (HRTEM) image (c) of the as-synthesized $Pd_{50}Cu_{50}$ nanodendrites. (The XRD peaks located between the Pd and Cu standard values and showed no single Pd and Cu peaks, which indicated the as-synthesized $Pd_{50}Cu_{50}$ nanodendrites were alloy structure. Scale bar: a is 200 nm, c is 5 nm.)

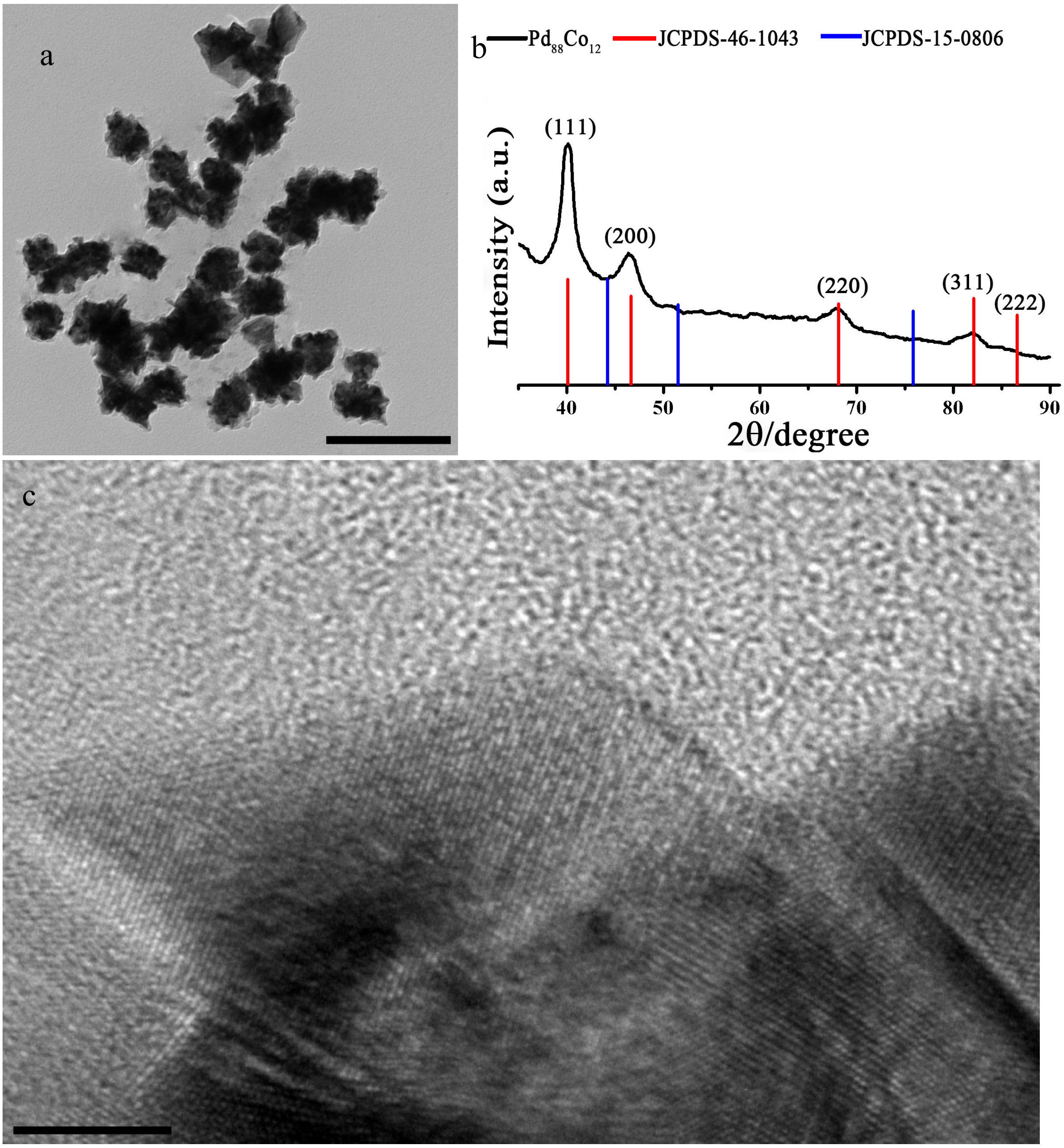

Supplementary Figure 3 | The transmission electron microscopy (TEM) image (a), The X-ray diffraction (XRD) pattern (b) and high-resolution TEM (HRTEM) image (c) of the as-synthesized $Pd_{88}Cu_{12}$ nanodendrites. (The XRD peaks located between the Pd and Co standard values and showed no single Pd and Co peaks, which indicated the as-synthesized $Pd_{88}Cu_{12}$ nanodendrites were alloy structure. Scale bar: a is 200 nm, c is 5 nm.)

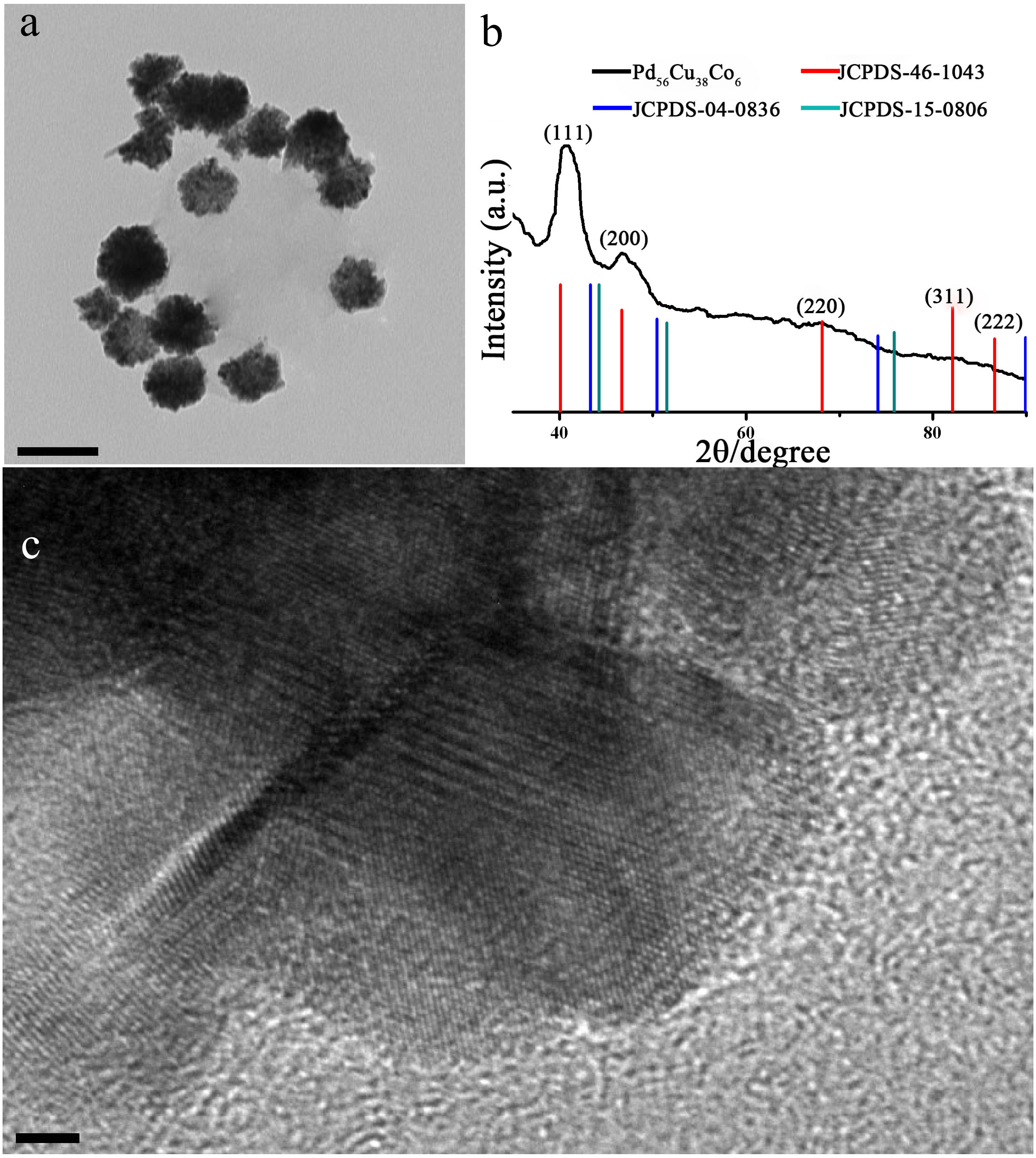

Supplementary Figure 4 | The transmission electron microscopy (TEM) image (a), The X-ray diffraction (XRD) pattern (b) and high-resolution TEM (HRTEM) image (c) of the as-synthesized $Pd_{56}Cu_{38}Co_6$ nanodendrites. (The XRD peaks located between the Pd, Cu and Co standard values and showed no single Pd, Cu and Co peaks, which indicated the as-synthesized $Pd_{56}Cu_{38}Co_6$ nanodendrites were alloy structure. Scale bar: a is100 nm, c is 2 nm.)

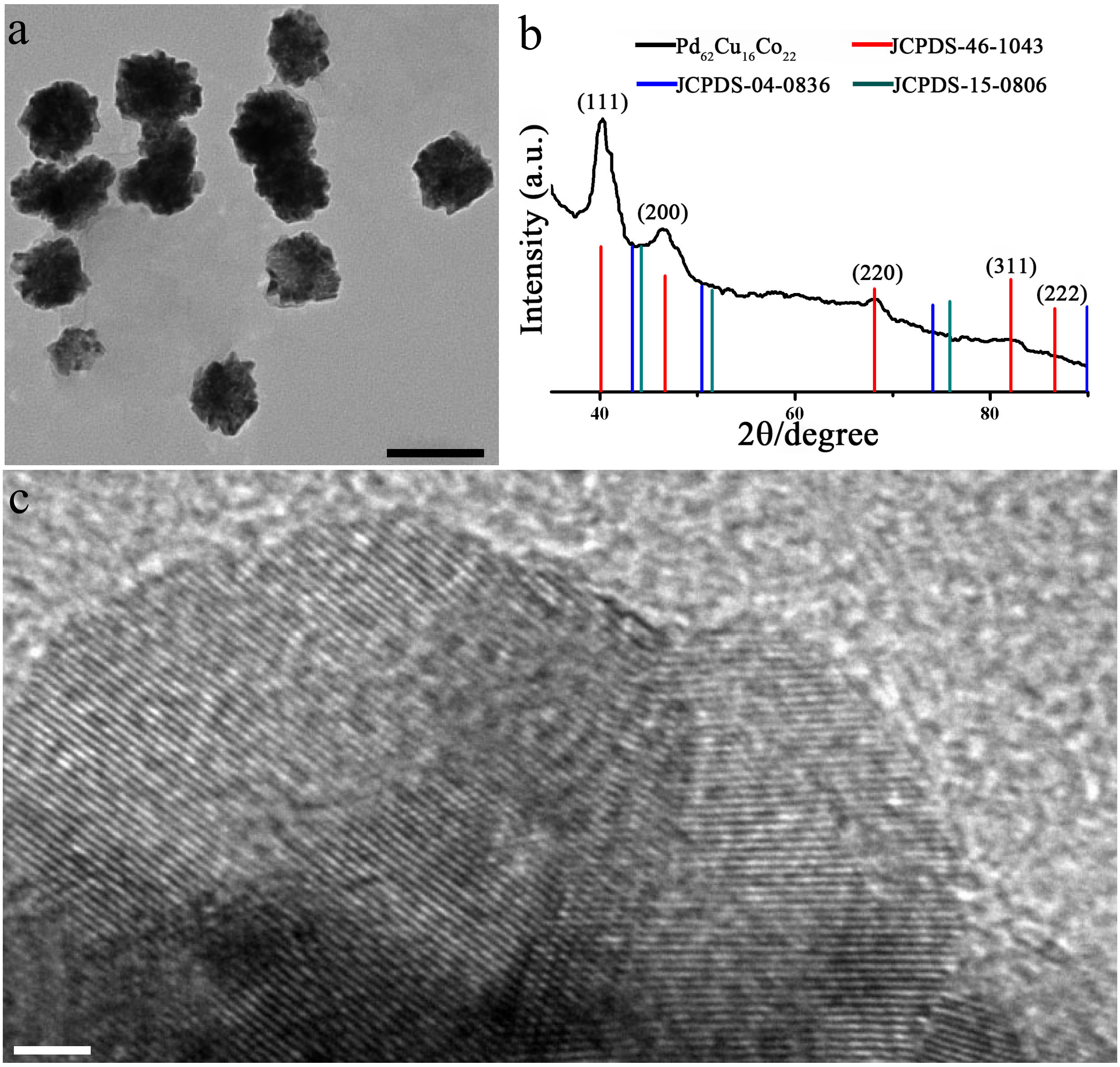

Supplementary Figure 5 | The transmission electron microscopy (TEM) image (a), The X-ray diffraction (XRD) pattern (b) and high-resolution TEM (HRTEM) image (c) of the as-synthesized $Pd_{62}Cu_{16}Co_{22}$ nanodendrites. (The XRD peaks located between the Pd, Cu and Co standard values and showed no single Pd, Cu and Co peaks, which indicated the as-synthesized $Pd_{62}Cu_{16}Co_{22}$ nanodendrites were alloy structure. Scale bar: a is100 nm, c is 2 nm.)

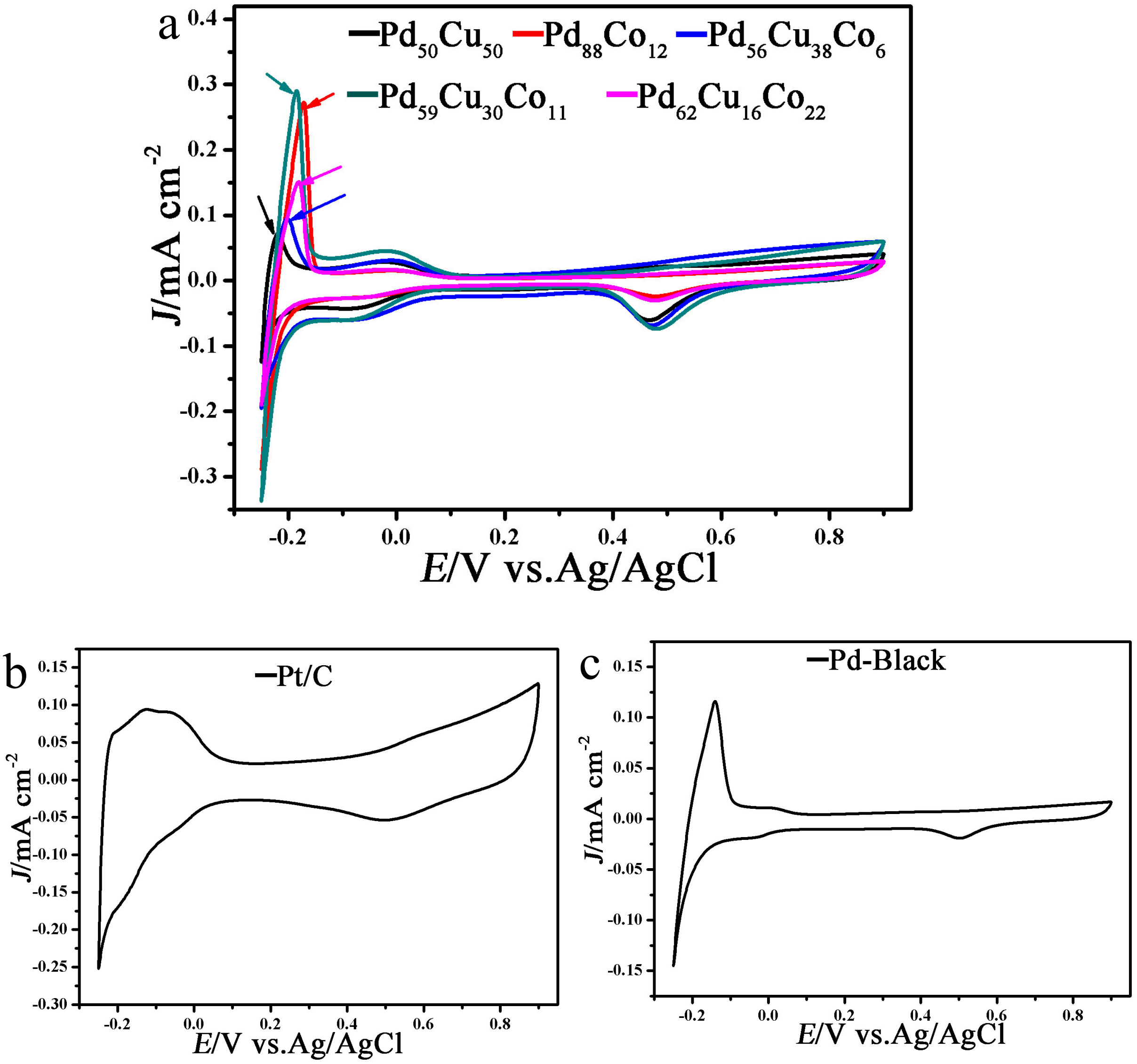

Supplementary Figure 6 | Cyclic voltammograms (CV) of the as-synthesized $Pd_{50}Cu_{50}$, $Pd_{88}Co_{12}$, $Pd_{56}Cu_{38}Co_{6}$, $Pd_{59}Cu_{30}Co_{11}$ and $Pd_{62}Cu_{16}Co_{22}$ (a), (b) Pt/C and Pd-Black (c) in 0.1 mol L^{-1} (M) $HClO_4$ solution at a rate of 50 mV s^{-1}.

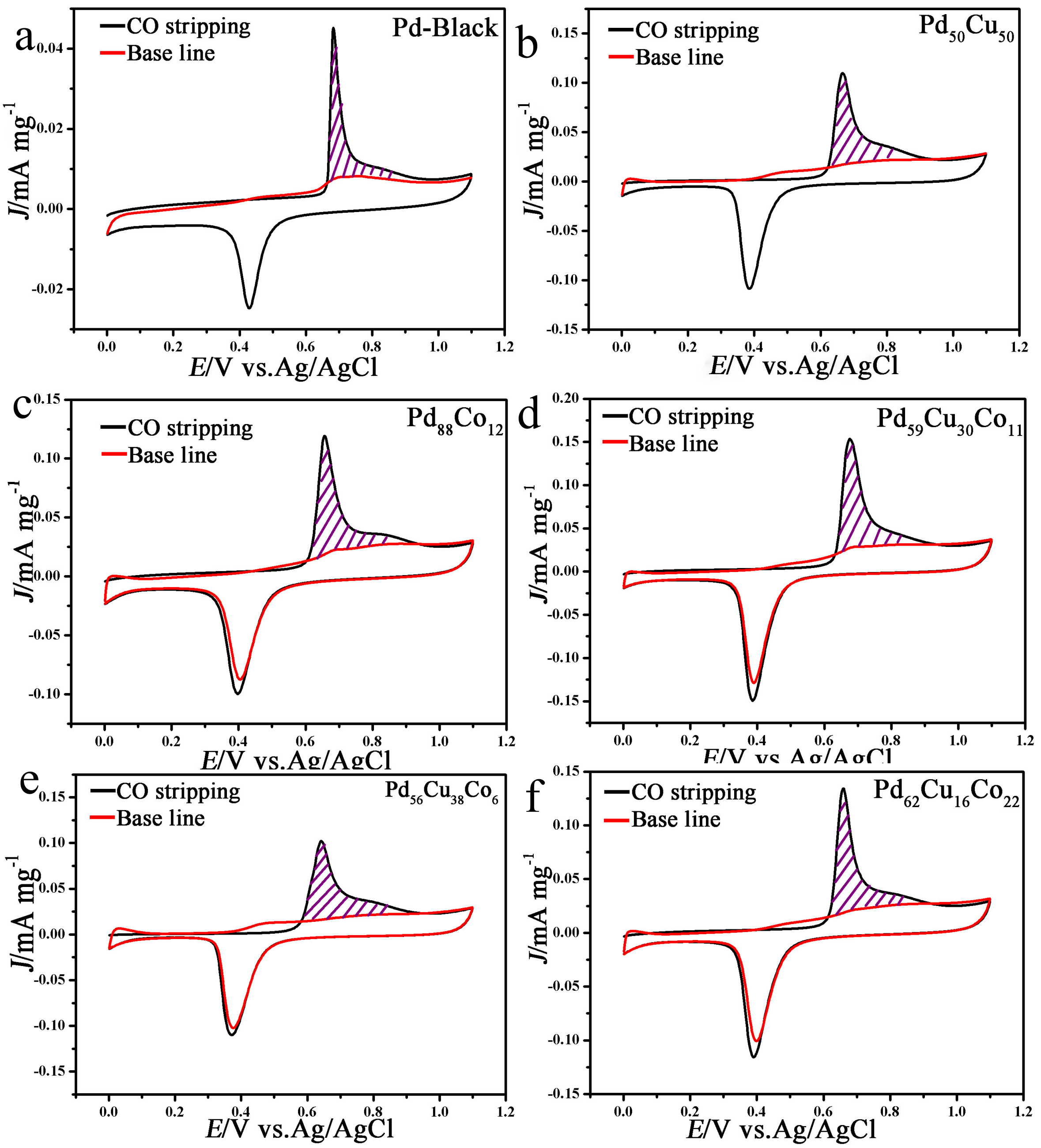

Supplementary Figure 7 | CO-stripping curves of Pd-Black (a), the as-synthesized $Pd_{50}Cu_{50}$ (b), the as-synthesized $Pd_{88}Co_{12}$ (c), the as-synthesized $Pd_{59}Cu_{30}Co_{11}$ (d), the as-synthesized $Pd_{56}Cu_{38}Co_{6}$ (e) and the as-synthesized $Pd_{62}Cu_{16}Co_{22}$ (f) (Black line is the CO stripping and the red line is base line).

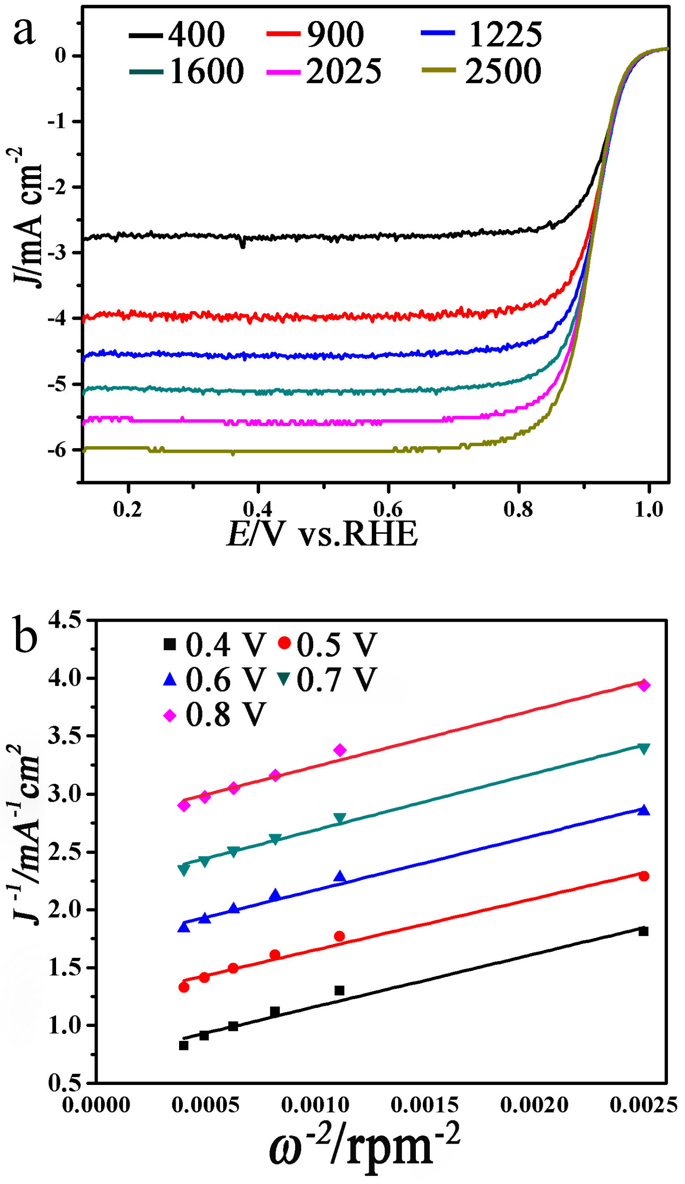

Supplementary Figure 8 | (a) The oxygen reduction reaction (ORR) polarization curves of $Pd_{59}Cu_{30}Co_{11}$ nanoalloys at different rotating rates. (b) The corresponding Koutecky-Levich plots at different potentials.

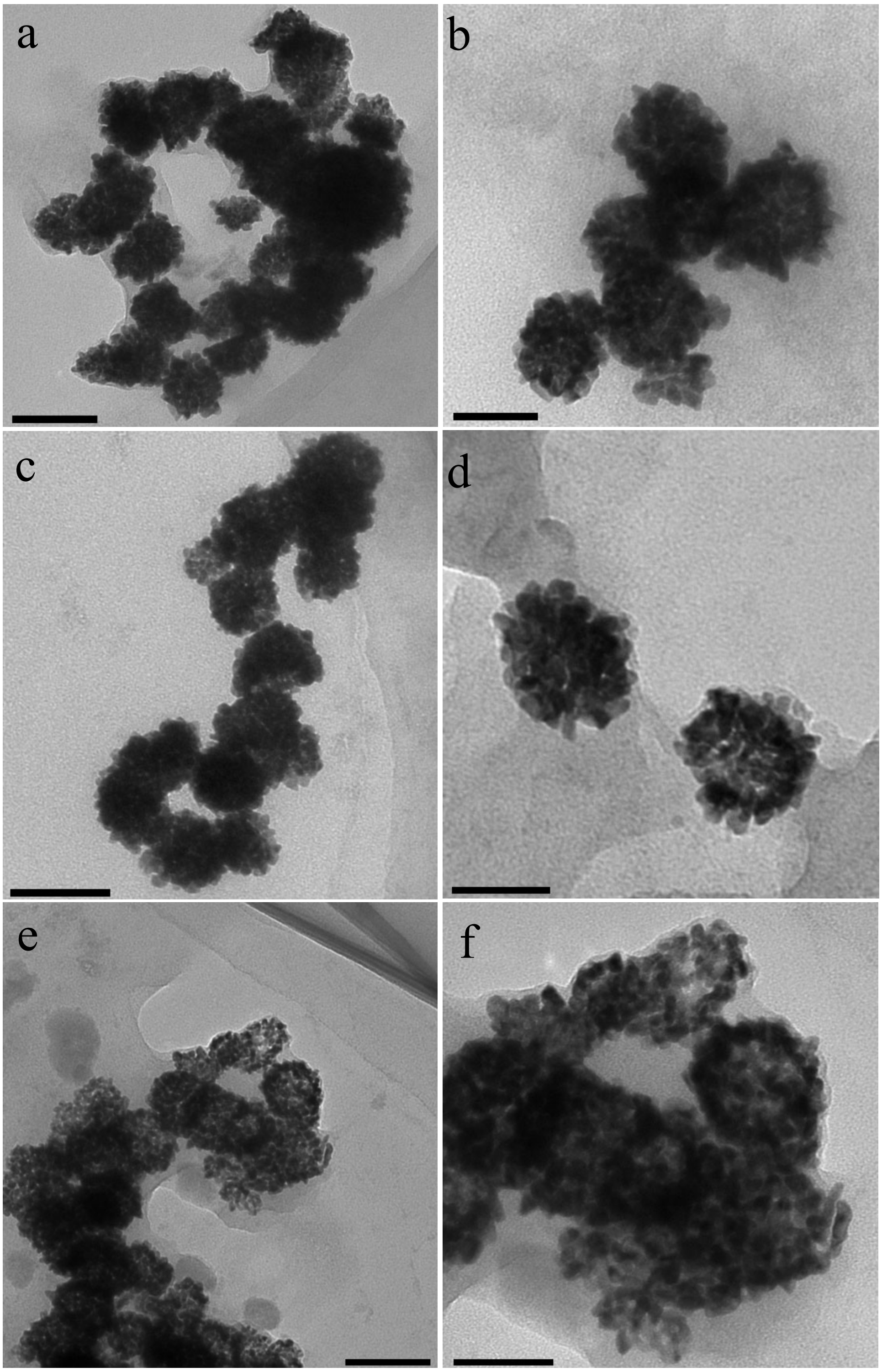

Supplementary Figure 9 | The transmission electron microscopy (TEM) images of the dendritic $Pd_{59}Cu_{30}Co_{11}$ nanoalloys after different cycles of accelerated durability tests (ADTs). (a, b) 5000 cycles; (c, d) 10000 cycles and (e, f) 15000 cycles. Scale bar: a, c and e are 100 nm, b, d and f are 50nm.

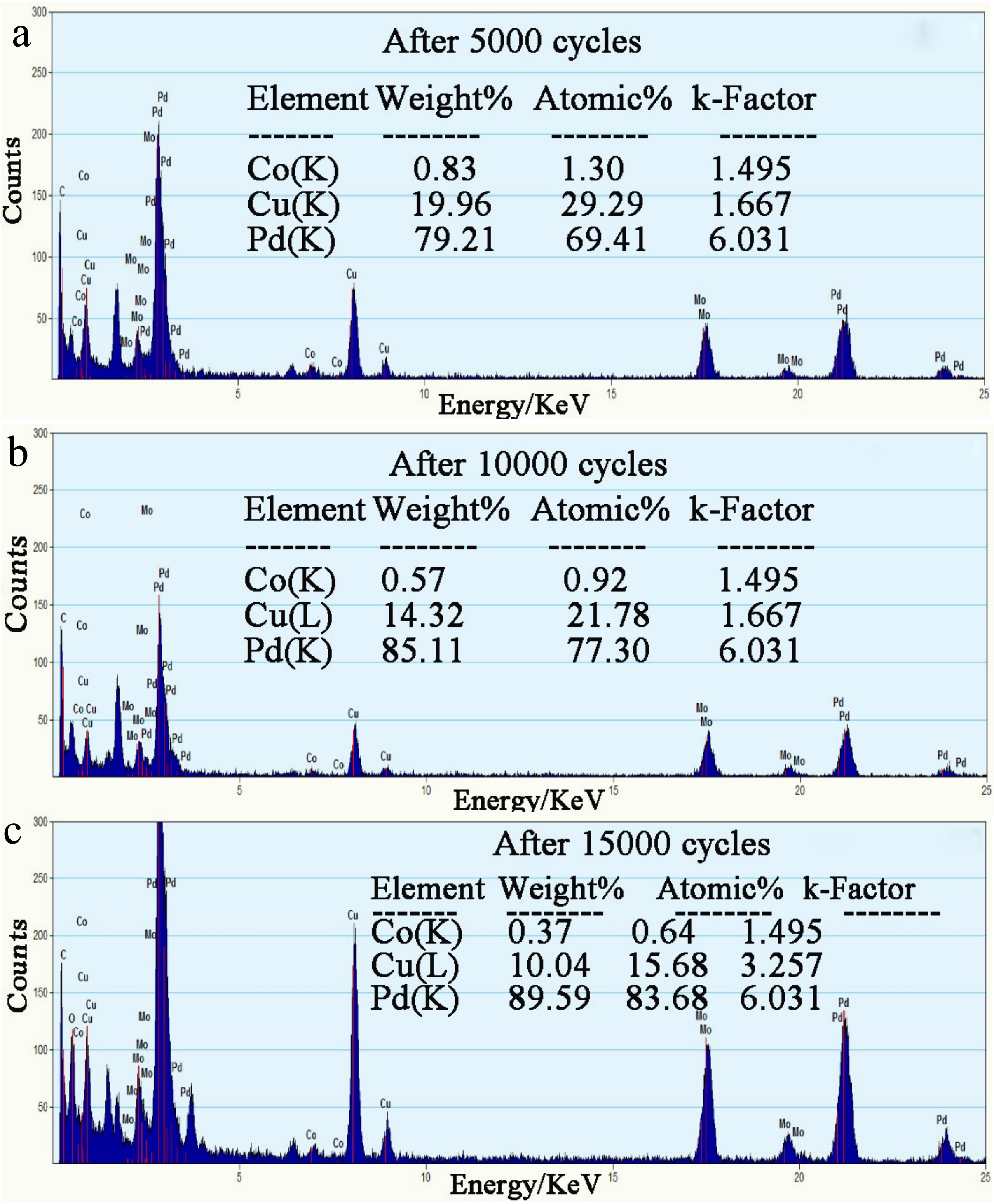

Supplementary Figure 10 | The data of energy dispersive X-ray spectra (EDS) of the samples as shown in Supplementary Figure 9. (A) after 5000 cycles; (B) after 10000 cycles and (C) after 15000 cycles.

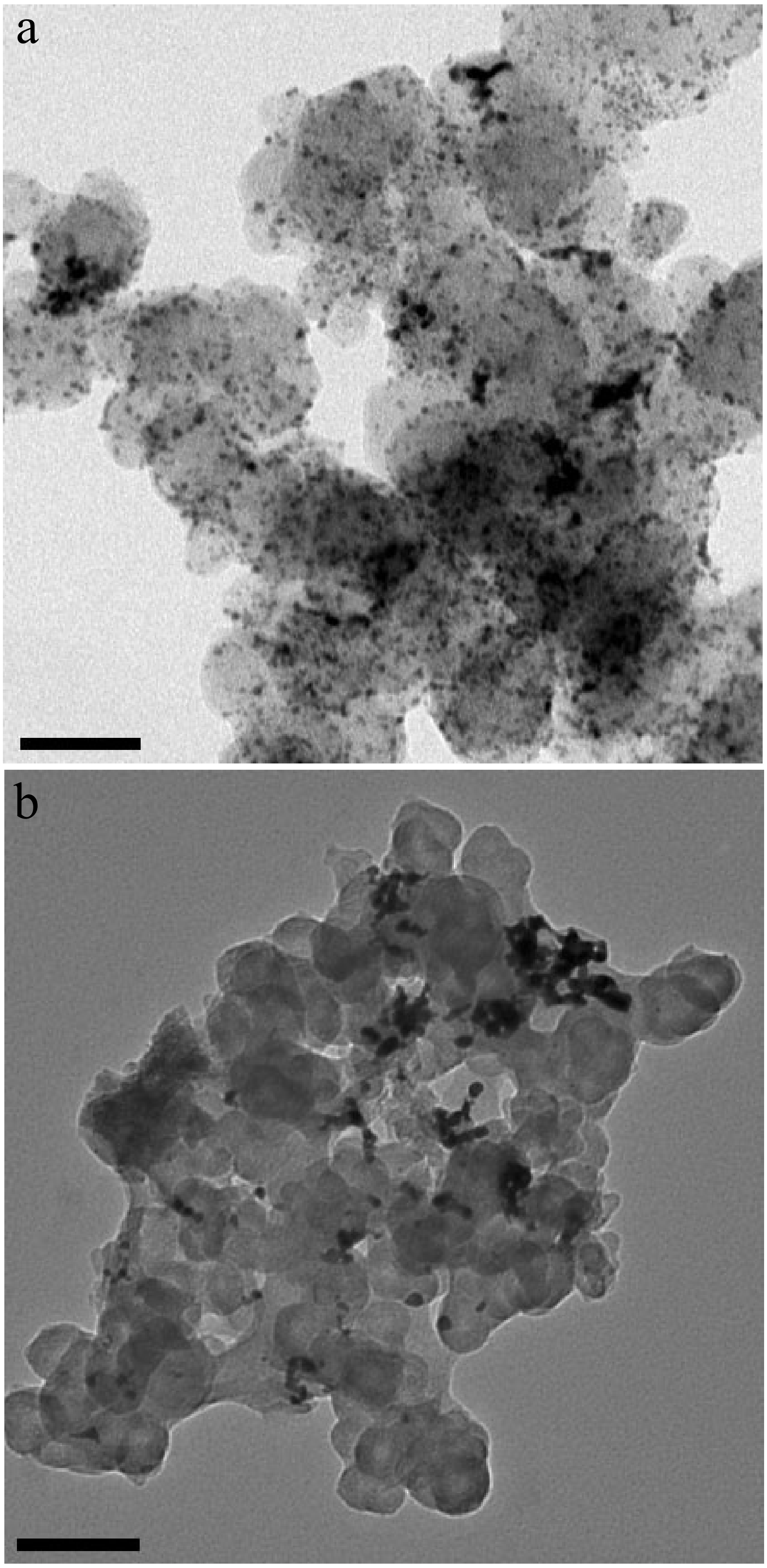

Supplementary Figure 11 | The transmission electron microscopy (TEM) images of commercial Pt/C. (a) Initial; (b) After 10000 cycles of accelerated durability tests (ADTs). Scale bar: a is 50 nm, b is 100 nm.

Supplementary Tables

Sample	Element	Atomic% Feeding radio	Atomic% (result from ICP-OES)
$Pd_{50}Cu_{50}$	Pd	50	50
	Cu	50	50
$Pd_{88}Co_{12}$	Pd	50	88
	Co	50	12
$Pd_{56}Cu_{38}Co_{6}$	Pd	50	56
	Cu	33.3	38
	Co	16.7	6
$Pd_{59}Cu_{30}Co_{11}$	Pd	50	59
	Cu	25	30
	Co	25	11
$Pd_{62}Cu_{16}Co_{22}$	Pd	50	62
	Cu	12.5	16
	Co	37.5	22

Supplementary Table 1 | The inductively coupled plasma optical emission spectrometer (ICP-OES) results of the as-synthesized $Pd_{50}Cu_{50}$, $Pd_{88}Co_{12}$, $Pd_{56}Cu_{38}Co_{6}$, $Pd_{59}Cu_{30}Co_{11}$ and $Pd_{62}Cu_{16}Co_{22}$.

Catalysts	Test Condition	Specific Activity (mA/cm^2)	Mass Activity (A/mg)	Reference
Ag-Co Nanoparyicle	0.1M KOH; Scan rate:10mV·s^{-1}	5.1 (0.8 V vs RHE)	NA	1
Rh_3Sn_1/C Nanoparticle	0.1M KOH; Scan rate:5mV·s^{-1}	3.6 (0.75 V vs RHE)	2.0 (0.8 V vs RHE)	2
Rh_8Pd_{92} Octahedron	0.1M $HClO_4$; Scan rate:10mV·s^{-1}	NA	0.26 (0.9 V vs RHE)	3
$Rh_{20}Pd_{80}$/C Nanodendrites	0.1M KOH; Scan rate:10mV·s^{-1}	NA	0.48 (0.8 V vs RHE)	4
PdCu B2 phase Nanoparticle	0.1M $HClO_4$; Scan rate:10mV·s^{-1}	0.168 (0.9 V vs RHE)	0.0415 (0.9 V vs RHE)	5
Pd tetrahedron /$W_{18}O_{49}$	0.1M KOH; Scan rate:10mV·s^{-1}	0.45 (0.9 V vs RHE)	0.216 (0.9 V vs RHE)	6
Nanoporous PdNi	0.1M KOH; Scan rate:10mV·s^{-1}	NA	0.4 (0.85 V vs RHE)	7
Pd-Co/gCN	1M H_2SO_4 Scan rate:10mV·s^{-1}	5.8 (0.65 V vs RHE)	NA	8
Cu_3Pd/Graphen e Nanoparticle	0.1 M KOH; Scan rate:50mV·s^{-1}	NA	0.045 (0.75 V vs RHE)	9
PdCuCo anisotropic structure	0.1M $HClO_4$; Scan rate:10mV·s^{-1}	0.252 (0.9 V vs RHE)<br>1.7 (0.8 V vs RHE)	0.178 (0.9 V vs RHE)<br>1.135 (0.8 V vs RHE)	10
$Pd_{0.90}Ni_{0.10}$ Nanowire	0.1M $HClO_4$; Scan rate:10mV·s^{-1}	1.96 (0.8 V vs RHE)	NA	11
Ordered PdCuCo/C Nanoparticle	0.1M NaOH; Scan rate:10mV·s^{-1}	NA	0.13 (0.9 V vs RHE)<br>0.37(0.875V vs RHE)	12
Pt/C Nanoparticle	0.1M KOH Scan rate:10mV.s^{-1}	0.41 ± 0.02 (0.9 V vs RHE)<br>0.46 ± 0.07 (0.875 V vs RHE)<br>$2.56\ \pm0.05$(0.8 V vs RHE)	0.11 ± 0.01 (0.9 V vs RHE)<br>0.29 ± 0.05 (0.875 V vs RHE)<br>1.61 ± 0.05 (0.8 V vs RHE)	This work
$Pd_{59}Cu_{30}Co_{11}$ Nanodendrite	0.1M KOH Scan rate:10mV.s^{-1}	0.90 ± 0.01 (0.9 V vs RHE)<br>1.73 ± 0.07 (0.875 V vs RHE)<br>10.20 ± 0.18 (0.8 V vs RHE)	0.38 ± 0.01 (0.9 V vs RHE)<br>1.01 ± 0.04 (0.875 V vs RHE)<br>5.98 ± 0.11 (0.8 V vs RHE)	This work
$Pd_{56}Cu_{38}Co_6$ Nanodendrite	0.1M KOH Scan rate:10mV.s^{-1}	0.65 ± 0.003 (0.9 V vs RHE)	0.18 ± 0.02 (0.9 V vs RHE)	This work
$Pd_{62}Cu_{16}Co_{22}$ Nanodendrite	0.1M KOH Scan rate:10mV.s^{-1}	0.58 ± 0.01 (0.9 V vs RHE)	0.15 ± 0.01 (0.9 V vs RHE)	This work

Supplementary Table 2 | Summary of mass activities on non-Pt catalysts for oxygen reduction reaction (ORR).

Catalysts	Test Condition	ECSA (m^2/g)	Specific Activity (mA/cm^2)	Mass Activity (A/mg)	Reference
Pd/THPP Nanoparticle	0.1 M $HClO_4$ + 0.1 M FA Scan rate:50mV·s^{-1}	168	NA	4.65	13
Pd/CoP Nanoparticle	0.5 M H_2SO_4 + 0.5 M FA Scan rate:50mV·s^{-1}	72.89	3.79	2.7572	14
PdCu Nanotripod	0.5 M $HClO_4$ + 0.5 M FA Scan rate:50mV·s^{-1}	28.7	5.8	1.58	15
Pd tetrapod	0.5 M $HClO_4$ + 0.25 M FA Scan rate:50mV·s^{-1}	15.9	3.84	NA	16
PdNi nanowire	0.5 M H_2SO_4 + 0.5 M FA Scan rate:50mV·s^{-1}	NA	NA	0.82	17
Pd Concave	0.5 M H_2SO_4 + 2 M FA Scan rate:50mV·s^{-1}	NA	11	NA	18
Pd Nanoparticle	0.1 M H_2SO_4 + 0.1 M FA Scan rate:50mV·s^{-1}	122	NA	3.39	19
Pd Nanosheet	0.5 M H_2SO_4 + 0.25 M FA Scan rate:50mV·s^{-1}	67	NA	1.38	20
PdCo Nanoparticle	0.1 M $HClO_4$ + 2 M FA Scan rate:50mV·s^{-1}	NA	NA	0.774	21
Pd Nanoparticle	0.5 M H_2SO_4 + 0.5 M FA Scan rate:50mV·s^{-1}	147.1	NA	0.1401	22
Pd Nanosheet	0.1 M $HClO_4$ + 0.2 M FA Scan rate:50mV·s^{-1}	35.6	NA	0.6343	23
PdCu Nanosheet	0.5 M H_2SO_4 + 0.25 M FA Scan rate:50mV·s^{-1}	137.6	NA	1.6283	24
Pd Black Nanoparticle	0.1 M $HClO_4$ + 0.5 M FA Scan rate:50mV·s^{-1}	17.1 ± 2.17	2.24±0.04	0.39±0.03	This work
Pt/C Nanoparticle	0.1 M $HClO_4$ + 0.5 M FA Scan rate:50mV·s^{-1}	67.8± 2.38	0.56±0.12	0.35±0.02	This work
$Pd_{50}Cu_{50}$ Nanodendrite	0.1 M $HClO_4$ + 0.5 M FA Scan rate:50mV·s^{-}	37.9± 2.93	6.38±0.28	2.42±0.11	This work
$Pd_{88}Cu_{12}$ Nanodendrite	0.1 M $HClO_4$ + 0.5 M FA Scan rate:50mV·s^{-}	41.2± 3.05	2.94±0.12	1.21±0.05	This work
$Pd_{56}Cu_{38}Co_6$ Nanodendrite	0.1 M $HClO_4$ + 0.5 M FA Scan rate:50mV·s^{-1}	46.3± 2.62	7.25±0.31	3.36±0.14	This work
$Pd_{59}Cu_{30}Co_{11}$ Nanodendrite	0.1 M $HClO_4$ + 0.5 M FA Scan rate:50mV·s^{-1}	58.7± 3.16	9.06±0.27	5.32±0.16	This work
$Pd_{62}Cu_{16}Co_{22}$ Nanodendrite	0.1 M $HClO_4$ + 0.5 M FA Scan rate:50mV·s^{-1}	44.7± 2.87	6.46±0.28	2.89±0.12	This work

Supplementary Table 3 | Summary of mass activities on Pd-based catalysts for formic acid oxidation (FAO).

Supplementary References

1. Holewinski, A. Idrobo, J. C. Linic, S. High-performance Ag-Co alloy catalysts for electrochemical oxygen reduction. *Nat. Chem.* **6**, 828-834 (2014).

2. Ahn, M. *et al*. Rhodium−tin binary nanoparticle-a strategy to develop an alternative electrocatalyst for oxygen reduction, *ACS Catal*. **7**, 5796-5801 (2017).

3. Yan, Y. *et al*. Kinetically-controlled growth of cubic and octahedral Rh-Pd alloy oxygen reduction electrocatalysts with high activity and durability. *Nanoscale* **7**, 301-307 (2015).

4. Qi, Y. Wu, J. Zhang, H. Jiang, Y. Jin, C. Fu, M. Yang, H. Yang, D. Facile synthesis of Rh-Pd alloy nanodendrites as highly active and durable electrocatalysts for oxygen reduction reaction. *Nanoscale* **6**, 7012-7018 (2014).

5. Wang, C. Chen, D. P. Sang, X. Unocic, R. R. Skrabalak, S. E. Size-dependent disorder−order transformation in the synthesis of monodisperse intermetallic PdCu nanocatalysts. *ACS nano.* **10**, 6345-6353 (2016).

6. Lu, Y. Jiang, Y. Gao, X. Wang, X. Chen, W. Strongly coupled Pd nanotetrahedron/tungsten oxide nanosheet hybrids with enhanced catalytic activity and stability as oxygen reduction electrocatalysts. *J Am. Chem. Soc.* **136**, 11687-11697 (2014).

7. Chen, L. *et al.* Nanoporous PdNi bimetallic catalyst with enhanced electrocatalytic performances for electro-oxidation and oxygen reduction reactions. *Adv. Funct. Mater.* **21,** 4364-4370 (2011).

8. Ghosh, A. Chandran, P. Ramaprabhu, S. Palladium-nitrogen coordinated cobalt alloy towards hydrogen oxidation and oxygen reduction reactions with high catalytic activity in renewable energy generations of proton exchange membrane fuel cell. *Appl. Energy* **208**, 37-48 (2017).

9. Zheng, Y. *et al.* Component-controlled synthesis and assembly of Cu–Pd nanocrystals on graphene for oxygen reduction reaction. *ACS Appl. Mater. Inter.* **7**, 5347-5357 (2015).

10. Zuo, Y. *et al.* Atomic vacancies control of Pd-based catalysts for enhanced electrochemical performance. *Adv. Mater.* **30**, 1704171 (2018).

11. Liu, H. Koenigsmann, C. Adzic, R. R. and Wong, S. S. Probing ultrathin one-dimensional Pd–Ni nanostructures as oxygen reduction reaction catalysts. *ACS Catal.* **4**, 2544-2555 (2014).

12. Jiang, K. *et al.* Ordered PdCu-based nanoparticles as bifunctional oxygen-reduction and ethanol-oxidation electrocatalysts. *Angew. Chem. Int. Ed.* **55**, 9030-9035 (2016).

13. Wang, X. Yang, J. Yi, H. Song, R. Tang, Z. "Raisin bun"-like nanocomposites of palladium clusters and porphyrin for superior formic acid oxidation. *Adv. Mater.* **25**, 2728-2732 (2013).

14. Feng, L. *et al*. Nanostructured palladium catalyst poisoning depressed by cobalt phosphide in the electro-oxidation of formic acid for fuel cells. *Nano Energy* **30**,355-361 (2016).

15. Zhang, L. *et al*. Pd–Cu bimetallic tripods: a mechanistic understanding of the synthesis and their enhanced electrocatalytic activity for formic acid oxidation. *Adv. Funct. Mater.* **24**, 7520-7529 (2015)

16. Dai, Y. *et al.* Carbon monoxide-assisted synthesis of single-crystalline Pd tetrapod nanocrystals through hydride formation. *J. Am. Chem. Soc.* **134**, 7073-7080 (2012).

17. Du, C. Chen, M. Wang, W. and Yin, G. Nanoporous PdNi alloy nanowires as highly active catalysts for the electro-oxidation of formic acid. *ACS Appl. Mater. Inter.* **3**, 105-109 (2011).

18. Jin, M. Zhang, H. Xie, Z. and Xia, Y. Palladium concave nanocubes with high-index facets and their enhanced catalytic properties. *Angew. Chem. Int. Ed.* **123**, 7850-7854 (2011).

19. Zhou, Z. Kang, X. Song, Y. and Chen, S. Butylphenyl-functionalized palladium nanoparticles as effective catalysts for the electrooxidation of formic acid. *Chem. Comm.* **47**, 6075-6077 (2011).

20. Huang, X. *et al.* Freestanding palladium nanosheets with plasmonic and catalytic properties, *Nat. Nanotech.* **6**, 28-32 (2011).

21. Mazumder, V. *et al*. More, K. L. Sun, S. A facile synthesis of MPd (M = Co, Cu) nanoparticles and their catalysis for cormic acid oxidation. *Nano Lett.* 12, 1102-1106 (2012).

22. Guo, C. X. Zhang, L.Y. Miao, J. Zhang, J. Li, C. M. DNA-functionalized graphene to guide growth of highly active Pd nanocrystals as efficient electrocatalyst for direct formic acid fuel cells. *Adv. Energy Mater.* **3**, 167-171 (2013).
23. Zhang, Y. *et al.* Seedless growth of palladium nanocrystals with tunable structures: from tetrahedra to nanosheets, *Nano Lett.* **15**, 7519-7525 (2015).
24. Yang, N. *et al.* Synthesis of ultrathin PdCu alloy nanosheets used as a highly efficient electrocatalyst for formic acid oxidation. *Adv. Mater.* **29**, 1700769 (2017).
